# MiR-30a-5p activates the AKT signalling pathway by targeting PHTF2 to inhibit migration and EMT of gastric cancer

**DOI:** 10.1038/s41598-025-33375-y

**Published:** 2025-12-20

**Authors:** Fei Tu, Fengyuan He, Zhiyuan Li, Yiqing Jia, Lingzhu Wang, Tiesuo Zhao, Sheng Guo, Yan Jin, Zhijun Yang

**Affiliations:** 1 School of Forensic Medicine, Xinxiang Medical University, Xinxiang, China; 2 Xinxiang Engineering Technology Research Center of Immune Checkpoint Drug for Liver-Intestinal Tumors, Xinxiang Medical University, Xinxiang, China; 3 School of Basic Medical Sciences, Xinxiang Medical University, Xinxiang, China

**Keywords:** Cancer, Cell biology, Molecular biology

## Abstract

MicroRNAs (miRNAs) play a very important role in the development of gastric cancer (GC). MiR-30a-5p Participates in the formation and progression of various cancers. However, the role and clinical value of miR-30a-5p in GC remain unclear. The expression of miR-30a-5p in GC cells and Gastric Epithelial Strain-1 (GES-1) was detected by quantitative real-time PCR (qPCR). Wound healing assay, transwell assay and western blot analyses were used to examined the effects of miR-30a-5p on GC cells in vitro. In silico prediction, qRT-PCR, dual luciferase reporter assays and western blot were applied to confirm the target genes of miR-30a-5p. The results indicated that miR-30a-5p inhibited the migration and Epithelial-Mesenchymal Transition (EMT) of GC cells by activating the AKT signalling pathway. Putative homeodomain transcriptional factor2 (PHTF2) was identified to be a direct target of miR-30a-5p. Knockdown of PHTF2 also suppressed the migration and EMT of GC cells, while overexpression of PHTF2 could promote the migration and EMT of GC cells and impede the AKT signalling pathway. miR-30a-5p can suppress the migration and EMT of GC cells by directly targeting PHTF2. Hence, miR-30a-5p may be a potential target for GC treatment.

**Supplementary Information:**

The online version contains supplementary material available at 10.1038/s41598-025-33375-y.

## Introduction

Gastric cancer (GC) is a prevalent malignancy of the digestive system. It is the fifth common cause of global cancer incidence in 2022, with an estimated almost one million new cases, occupying 4.9% of all cancer cases. Meanwhile, it is also the fifth leading cause of cancer-related mortality across the globe, with 660,000 deaths (6.8% of all cancer deaths)^1^. Although researches have made great developments in GC screening, diagnosis, and treatment, the overall 5-year survival remained low^2^. The main cause of cancer-related death of GC patients is attributed to metastasis^3^. Migration of cancer cells is fundamental to the process of tumor metastasis. Thus, it is very important to elucidate the molecular mechanism underlying GC migration for identifying new therapeutic targets and providing new ideas for early diagnosis.

MicroRNAs (miRNAs) are a type of endogenous, single-stranded, small non-coding RNAs containing 18 ~ 24 nucleotides, which inhibit target messenger RNA (mRNA) 3’-untranslated region(UTR), resulting in the degradation of mRNAs or suppression of translation^4,5^. To date, hundreds of miRNA have been confirmed that they played vital roles in tumor proliferation, migration, invasion, and metastasis^6–8^. Over the past few years, expanding evidence has indicated that abnormally expressed miRNAs may represent either oncogenes or tumor suppressors to regulate the GC progression. For example, miR-144-3p inhibited gastric cancer progression by regulating GLI2^9^. miR-875-5p also suppressed proliferation, migration and invasion in GC cells via targeting USF2^10^. On the contrary, miR-135b-5p^11^, miR-1298-5p^12^, and miR-20a-5p^13^ have been confirmed as oncogenic factors that promoted gastric cancer development. Remarkably, previous research has demonstrated that dysregulation of miR-30a-5p has been studied in various diseases, consisting of hepatocellular carcinoma^14^, prostatic cancer^15^, ovarian cancer^16^, renal cell carcinoma^17^, head and neck cancer^18^. However, the molecular mechanism of miR-30a-5p in GC migration needs to be further explored.

In the present study, we screened candidate target genes of miR-30a-5p by RT-qPCR and dual luciferase reporter assay. *PHTF2* was identified as a direct target gene of miR-30a-5p. Then, this study aims to explore the biological role and regulatory mechanism of miR-30a-5p in gastric cancer cells. Here, we verified that miR-30a-5p inhibited the migration and EMT in GC cells by targetting PHTF2 via activating the AKT signalling pathway. The research results further provided a new gene target for the diagnosis and treatment of gastric cancer.

## Materials and methods

### Cell lines and cell culture

Four human GC cell lines (SGC-7901, MGC-803, HGC-27, AGS) and GES-1were purchased from the Type Culture Collection of the Chinese Academy of Sciences (Shanghai, China). Cells were maintained in Dulbecco’s Modified Eagle medium (DMEM; Gibco, USA), supplemented with 10% fetal bovine serum (FBS, WISENT CORPORATION), 100 U/mL penicillin, and 100 µg/mL streptomycin. All cells were incubated in a humidified atmosphere containing 5% CO_2_ at 37 °C.

### Oligonucleotide transfection

miR-30a-5p mimics, inhibitor, siPHTF2(si429, si1485) and their negative control (NC) were all synthesized by GenePharma (Shanghai, China). Oligonucleotide transfection was performed using RFect siRNA/miRNA Transfection Reagent (Cat#:11013, Baidai biotechnology, Changzhou, China) according to the manufacturer’s protocol. The sequences were listed in Table 1.

**Table 1:** Oligonucleotide sequences.

Name^a^	Sequence (5′ to 3′)^b^	Usage
miR-30a-5p mimics	UGUAAACAUCCUCGACUGGAAG	Transfection
Inhibitor (sense)	CUUCCAGUCGAGGAUGUUUACA	Transfection
si429 (sense)	CUGGCUUCUUGUCCUUUAUTT	Transfection
si1485 (sense)	CAGCCAUAUACCAGGAAUATT	Transfection
U6-F	CTCGCTTCGGCAGCACA	qRT-PCR
U6-R	AACGCTTCACGAATTTGCGT	qRT-PCR
PHTF2-F	ATGGCGTCCAAAGTCACAGA	qRT-PCR
PHTF2-R	CTACATTATACCCACCTGTT	qRT-PCR
miR-30a-5p-RT	GTCGTATCCAGTGCAGGGTCCGAGGTATTCGCACTGGATACGACCTTCCA	RT
miR-30a-5p-Q	CGCGTGTAAACATCCTCGAC	qRT-PCR
GAPDH-F	AAATCCCATCACCATCTTCC	qRT-PCR
GAPDH-R	TCACACCCATGACGAACA	qRT-PCR
PHTF2-wt-F	tgtttaaacgagctcgctagcCAATTCAAAGAAAAGAAGATGTAGCC	plasmid construction
PHTF2-wt-R	cttgcatgcctgcaggtcgacCACAATTTGTTATTTTGCCAATTATTT	plasmid construction
PHTF2-mut-F	TGAAG*cagcgct*ATCAGACTGTCTTGTGCAATTCTTATATT	mutagenesis
PHTF2-mut-R	CTGAT*agcgctg*CTTCAACTCCCTAGCTGAGACTCC	mutagenesis
PHTF2-F	cttggtaccgagctcggatccATGGCGTCCAAAGTCACAGATG	plasmid construction
PHTF2-R	aacgggccctctagactcgagTCATGACTTAATCTTCCATAGCTTTAAA	plasmid construction

^a^F, forward primer; R, reverse primer.

^b^Mutated target sites are underlined.

### RNA extractions and qRT-PCR

Total RNA was extracted from GC cell lines using TRIzol reagent (Invitrogen, CA, USA) according to the manufacturer’s instructions. Then we used FastKing RT Kit (With gDNase) (TIANGEN, China) to synthesize cDNA. qRT-PCR was performed on a Quant Studio5 Real-time PCR System (Applied Biosystems, USA) using ChamQ Universal SYBR qPCR Master Mix (Vazyme Biotech, Nanjing, China).

### MiRNA expression

miRNA expression was quantified using miRNA Universal SYBR^®^ qPCR Master Mix Assays (Vazyme Biotech, Nanjing, China). Reverse transcription was carried out with the miRNA 1st Strand cDNA Synthesis Kit (by stem-loop) (Vazyme Biotech, Nanjing, China) following the manufacturer’s instructions. Quantitative real-time PCR was performed on a Quant Studio5 Real-time PCR System (Applied Biosystems, USA). Amplification data were normalised to endogenous U6 expression. All procedures were carried out in triplicate and relative expression was calculated by the 2^−ΔΔCT^ method.

### Plasmid construction and dual-luciferase assay

The fragment of the 3′ UTR of *PHTF2* containing the predicted miR-30a-5p binding site was amplified by PCR and inserted between the NheI and SalI restriction sites of the pmirGLO Dual-Luciferase miRNA Target Expression Vector (kindly provided by Prof. Qifa Li of Nanjing Agricultural University, Nanjing, China). For mutation, miR-30a-5p binding motif in the 3’UTR of *PHTF2* gene was mutated by using the Mut Express MultiS Fast Mutagenesis Kit V2 (Vazyme Biotech, Nanjing, China). Luciferase activity was assessed 24 h post-transfection using the Dual-Glo Luciferase Detection System (Promega), with Renilla luciferase activity as an internal control. In addition, *PHTF2* CDS was amplified by PCR and cloned into pcDNA3.1(+) (also provided by Prof. Li) by ClonExpress Ultra One Step Cloning Kit V3(Vazyme Biotech, Nanjing, China). All plasmid constructs were sequence-verified.

### Protein extraction and Western blotting

The cell pellets were harvested and re-suspended using lysis buffer (20 mM Tris–HCl, pH 7.4, 150 mM NaCl, 1% Triton X-100, 25 mM β-glycerol-phosphate, 1 mM Na_3_VO_4_, 10% glycerol, 1X PMSF, with the Sigma phosphatase inhibitors and protease inhibitor (Pierce, Rockford, IL, USA)). The re-suspended cell pellet was then incubated on ice for 20 min, followed by centrifugation at 12,000×*g* for 20 min at 4 °C. The supernatants were collected and protein concentrations were measured using the BCA Protein Assay Kit (Beyotime, Shanghai, China). The lysates were separated by SDS-PAGE and transferred to PVDF membranes. Membranes were blocked with 5% non-fat milk in TBST at room temperature for 2 h, and incubated with primary antibodies in TBST overnight at 4 °C. Subsequently, the membranes were incubated with HRP-conjugated secondary antibodies for 2 h at room temperature. The protein bands were detected by the BeyoECL Star kit (Beyotime, Shanghai, China) and visualized using a multifunctional protein imaging system (Cell biosciences, USA). The following antibodies were used: β-actin (protein-tech,60008-1-Ig), GAPDH (protein-tech, 60004-1-Ig), PHTF2(Beijing Solarbio Science & Technology, K108037P), E-cadherin (Wanleibio, #WL01482),Vimentin (Santa Cruz, sc-6260), p-AKT(CST, #4058), AKT(CST, #4691). The bands were analyzed for gray-scale using ImageJ software. The gray-scale value of each target protein band was divided by the corresponding GAPDH or β-actin band’s gray-scale value to correct for differences in sample loading. The differences were compared through relative protein expression levels. All experiments were repeated three times.

### Scratch wound healing assay

The transfected cells were incubated into 12-well plates (2 × 10^5^ cells/well). After 24 h, the plate was scratched using a 200 µL pipette. Then the cells were washed and incubated with fresh serum-free medium. Images were acquired separately at 0 h and 48 h under an inverted microscope. Experiments were performed in triplicate. Images of the wound area were captured at 0 h and 48 h under an inverted microscope. The wound area was quantified using ImageJ software. The percentage of wound healing was calculated by the formula: (area at 0 h - area at 48 h) / area at 0 h × 100%. At least five random fields of view were selected for analysis in each group. The experiment was independently repeated three times.

### Transwell assay

For transwell migration assays, the transfected cells were plated in the upper chamber in medium without serum. A medium containing 10% FBS was put into the lower chamber as a stimulus. After wells were incubated for 24 h at 37 °C, the surface of cells on the upper membrane were removed. The cells were fixed and stained with 0.05% crystal violet. Six random fields of each chamber were photographed using an inverted microscope.

### Statistical analysis

The results are presented as means ± standard deviation. The statistical differences between groups were analyzed using t-tests of GraphPad Prism9.5. All experiments were performed at least in triplicate. P-values < 0.05 were considered to be statistically significant.

## Results

### miR-30a-5p inhibited migration and EMT of GC cells

**Fig. 1:**
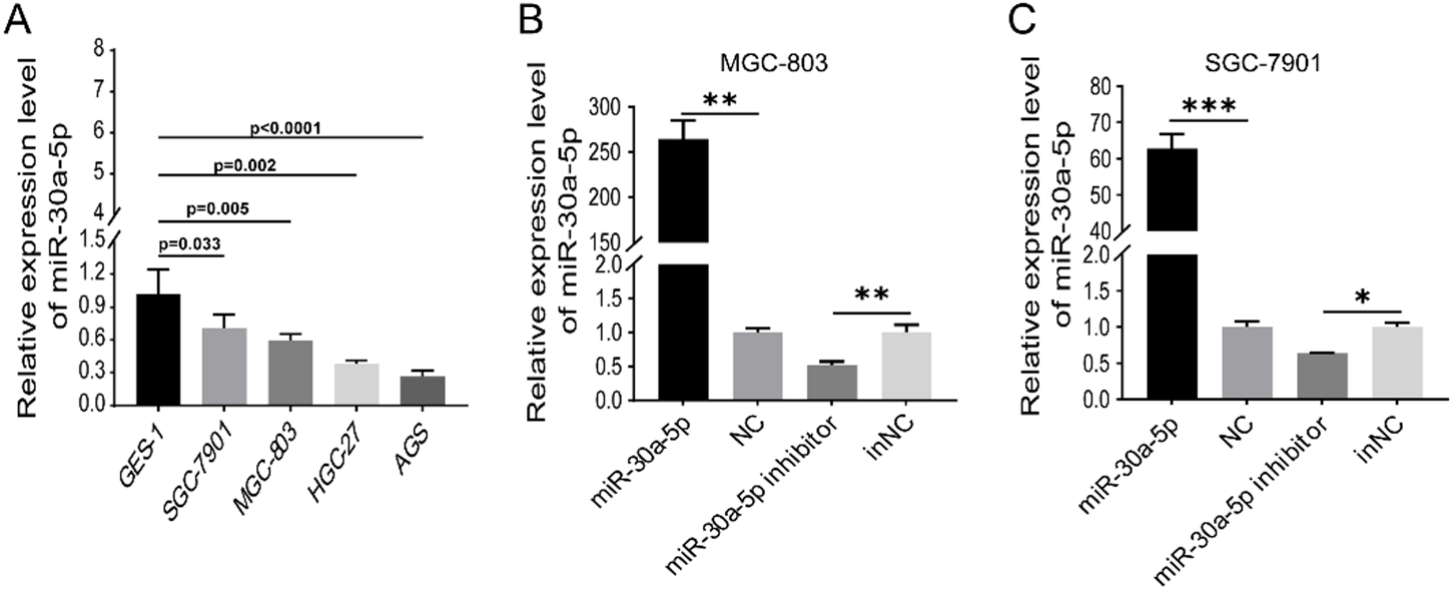
The expression level of miR-30a-5p in gastric cancer (GC) cells. (**A**) The expression level of miR-30a-5p in GC cell lines and Gastric Epithelial Strain-1 (GES-1) was measured by RT-qPCR. (**B**,**C**) The expression of miR-30a-5p in MGC-803 and SGC-7901 transfected with miR-30a-5p mimic and inhibitor respectively was measured by RT-qPCR. *p < 0.05, **p < 0.01, ***p < 0.001. *NC* negative control, *inNC* inhibitor negative control. The data expressed as the mean ± SD.

**Fig. 2:**
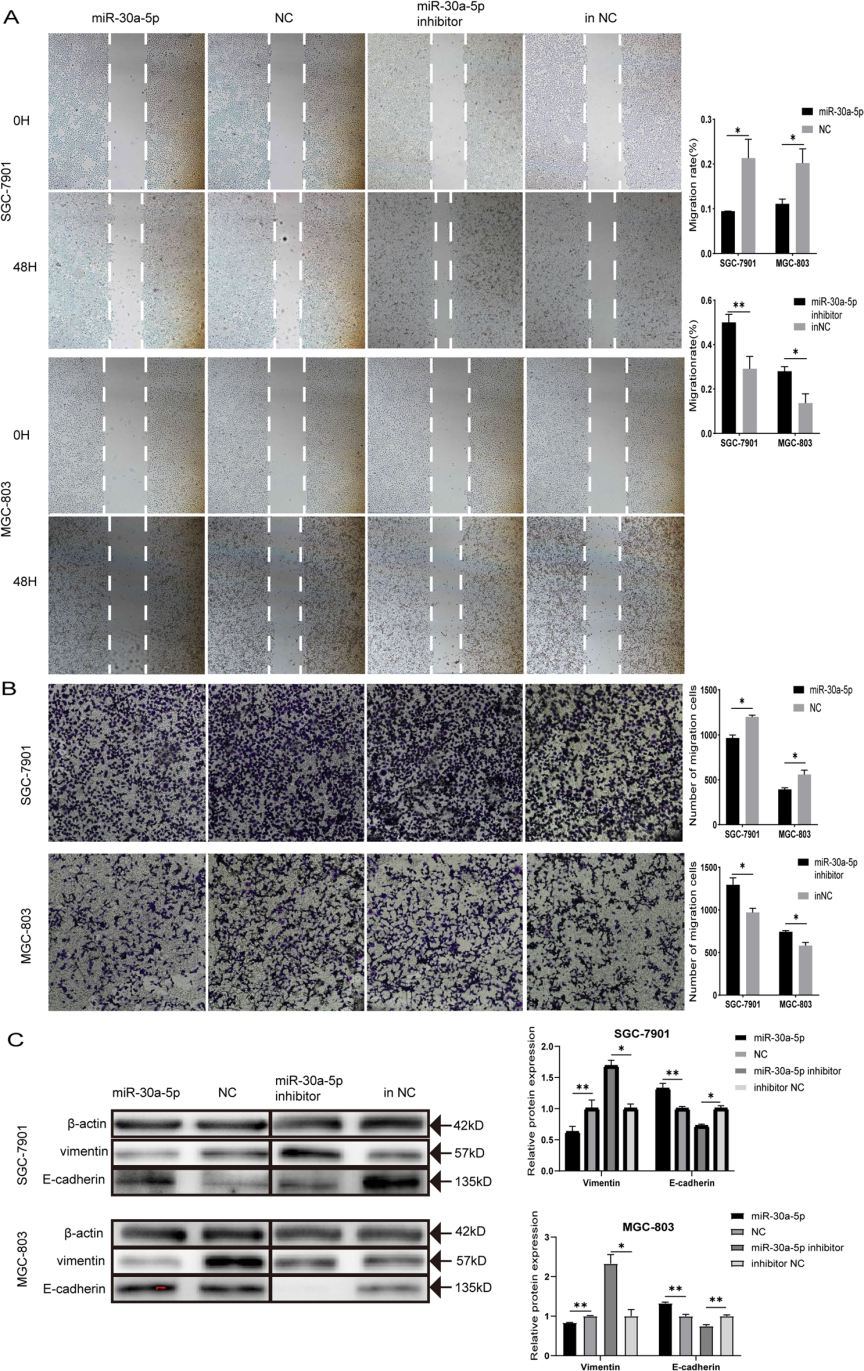
miR-30a-5p inhibited gastric cancer cell migration and Epithelial-Mesenchymal Transition (EMT). (**A**) The wound healing assays were performed to assess the effect of miR-30a-5p on cell motility at 0 and 48 H. (**B**) The transwell assays were performed to detect the effect of miR-30a-5p on migration. (**C**) The effect of miR-30a-5p on E-cadherin and vimentin protein expression in MGC-803 and SGC-7901 cells was detected by Western blot. *p < 0.05, **p < 0.01, ***p < 0.001. *NC* negative control, *inNC* inhibitor negative control. The data expressed as the mean ± SD.

To explore the role of miR-30a-5p in GC, we selected the SGC-7901 and MGC-803 cell lines for further study on the basis of the expression of miR-30a-5p in GC cell lines and GES-1 (Fig. 1A). Next, we overexpressed miR-30a-5p and silenced it in GC cells by transfecting mimic or inhibitor, respectively. RT-qPCR was used to detect the transfection efficiency of them (Fig. 1B,C). According to the wound healing assay results, the miR-30a-5p significantly impeded the migration of GC cell, while the miR-30a-5p inhibitor had the opposite effect (Fig. 2A). Similarly, the transwell assay results showed that overexpression of miR-30a-5p suppressed cell migration, and knockdown of miR-30a-5p promoted the migration of GC cells (Fig. 2B). To assess the effect of miR-30a-5p on EMT of GC cells, the markers of EMT were detected by western blotting. The results indicated that enhancement of miR-30a-5p intensified the expression level of epithelial marker E-cadherin, and weakened the vimentin in GC cells (Fig. 2C). Conversely, inhibition of miR-30a-5p reduced the E-cadherin and heightened the vimentin expression level (Fig. 2C). Collectively, these findings demonstrated that miR-30a-5p negatively regulated GC cell migration by hampered EMT process.

### PHTF2 was a direct target of miR-30a-5p

**Fig. 3:**
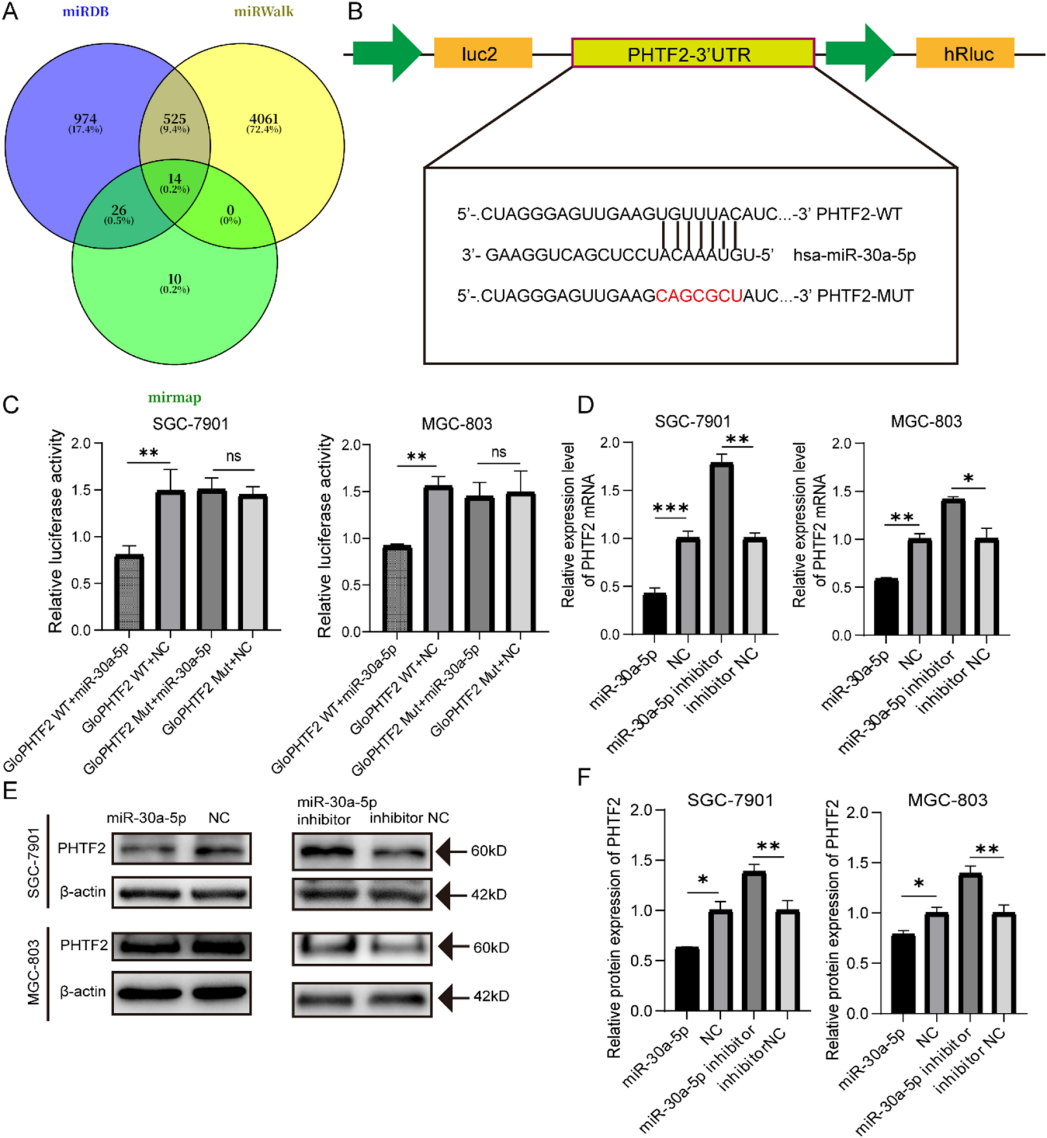
Putative homeodomain transcriptional factor2(PHTF2) was a direct target of miR-30a-5p in gastric cancer cells. (**A**) Putative targets of miR-30a-5p were predicted using the miRDB (https://mirdb.org), miRWalk (http://mirwalk.umm.uni-heidelberg.de), and miRmap (https://mirmap.ezlab.org) algorithms. (**B**) The 3’ UTR of PHTF2 mRNA contains the binding sequences of miR-30a-5p. (**C**) The dual- luciferase reporter assay confirmed the targeting relationship between miR-30a-5p and 3’ UTR of PHTF2 mRNA. (**D**) The effect of miR-30a-5p on PHTF2 mRNA expression level in MGC-803 and SGC-7901 cells was detected by RT-qPCR. (E-F) The effect of miR-30a-5p on PHTF2 protein expression in MGC-803 and SGC-7901 cells was detected by Western blot, and bar plots show the quantitative results of Western blot. *p < 0.05, **p < 0.01, ***p < 0.001, ns, not significant. *NC* negative control, *inNC* inhibitor negative control. The data expressed as the mean ± SD.

To investigate the underlying molecular mechanism of miR-30a-5p, putative miR-30a-5p target genes were confirmed by miRDB, miRWalk and mirmap. A total of 14 genes were overlapped (Fig. 3A). *PHTF2* was chosen as a potential target of miR-30a-5p based on literature about the 14 genes and preliminary experiments (Fig. S1). Next, we performed the RNAhybrid to speculate the binding site for miR-30a-5p in the 3’UTR of the *PHTF2* gene. The results illustrated a schematic representation of the wild-type (*PHTF2*-WT) and mutant (*PHTF2*-MUT) binding sites for miR-30a-5p within the 3’UTR sequence of *PHTF2* gene (Fig. 3B). To verify this possibility, we transfected SGC-7901 and MGC-803 cells with miR-30a-5p mimic, inhibitor and their negative controls. The results manifested that miR-30a-5p decreased in PHTF2 expression at the mRNA and protein levels but miR-30a-5p knockdown increased them (Fig. 3D–F). Subsequently, to determine whether miR-30a-5p could directly bind with 3’UTR of *PHTF2*, we co-transfected the *PHTF2*-WT or *PHTF2*-MUT with miR-30a-5p mimic or NC into SGC-7901 and MGC-803 cells. The dual luciferase reporter assays results uncovered that co-transfection with miR-30a-5p mimic observably lessened the firefly luciferase activity of the *PHTF2*-WT but not that of the *PHTF2*-MUT reporter plasmid (Fig. 3C). In summary, we concluded that *PHTF2* was a direct target of miR-30a-5p.

### PHTF2 regulated migration and EMT of GC cells

**Fig. 4:**
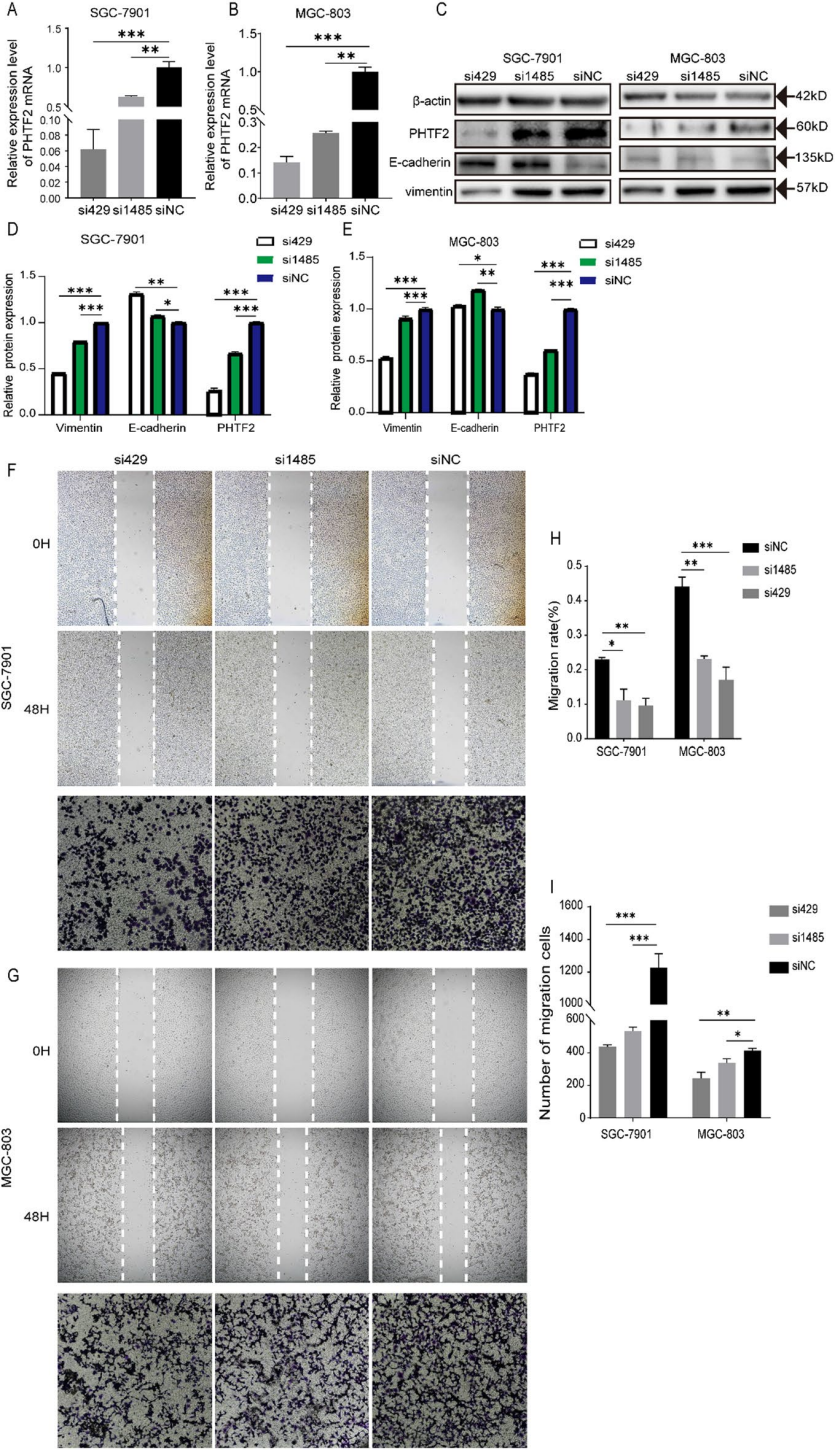
Knockdown of PHTF2 led to the inhibition of gastric cancer cell migration. (**A**,**B**) The expression of PHTF2 mRNA was measured by RT-qPCR. (**C**–**E**) The protein expression levels of PHTF2, E-cadherin and vimentin were measured by western blot, and bar plots show the quantitative results of Western blot. (**F**–**I**) Representative images of wound healing assay and transwell assay for SGC-7901 and MGC-803 cells (left panels) and bar plots show the quantitative results. *p < 0.05, **p < 0.01, ***p < 0.001. The data expressed as the mean ± SD.

**Fig. 5:**
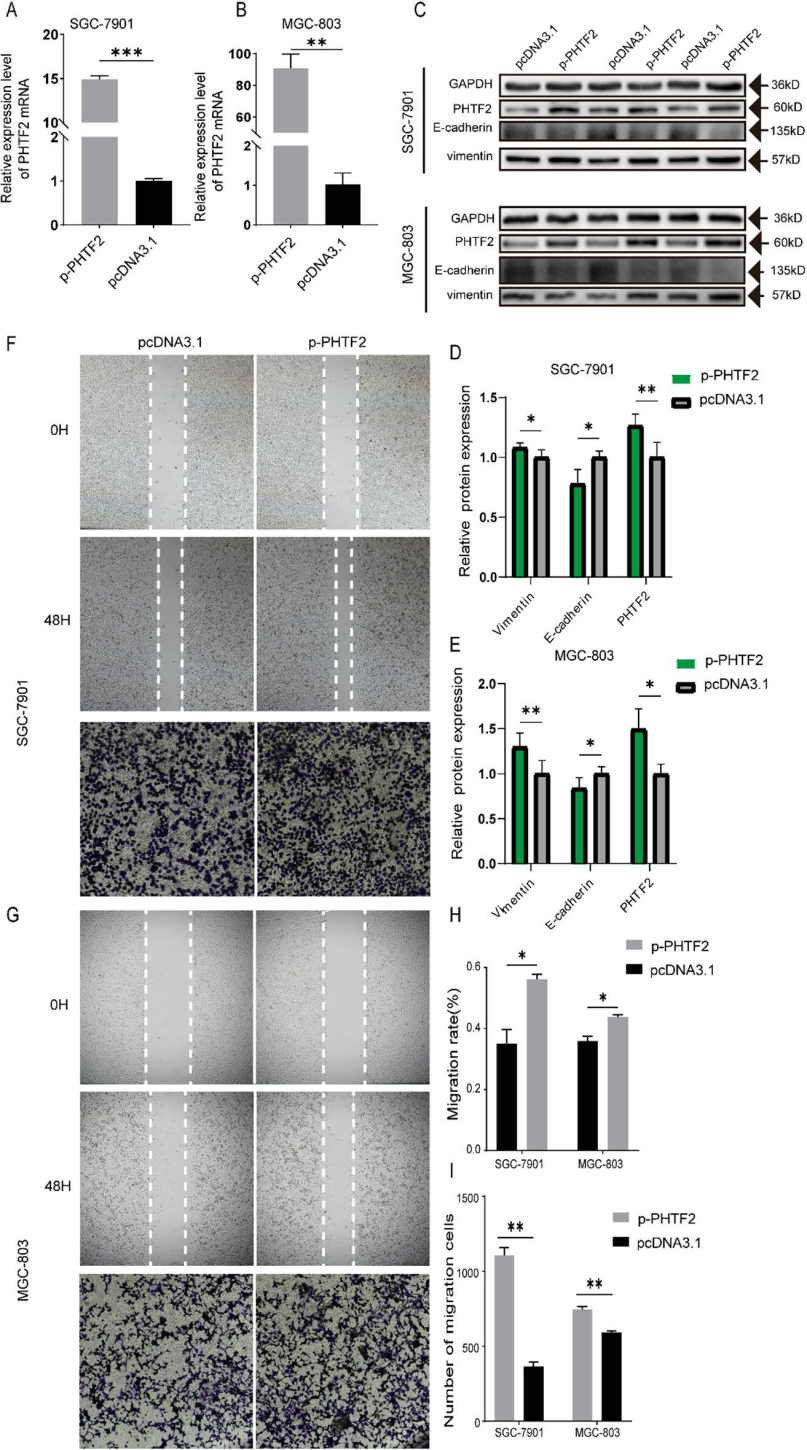
Overexpression of PHTF2 markedly promoted the migratory capacity of gastric cancer cells. (**A**,**B**) The expression of PHTF2 mRNA was measured by RT-qPCR. (**C**–**E**) The protein expression levels of PHTF2, E-cadherin and vimentin were measured by western blot, and bar plots show the quantitative results of Western blot. (**F**–**I**) Representative images of wound healing assay and transwell assay for SGC-7901 and MGC-803 cells (left panels) and bar plots show the quantitative results. *p < 0.05, **p < 0.01, ***p < 0.001. The data expressed as the mean ± SD.

To probe the impact of PHTF2 on the migration abilities, accompanied by the EMT of GC cells, first we knocked down *PHTF2* using siRNA constructs (si429 and si1485) in SGC-7901 and MGC-803 cells, and observed prominent decreases at the mRNA and protein levels (Fig. 4A–C). The wound healing assay and transwell assay results revealed that depletion of PHTF2 attenuated the abilities of migration in GC cells (Fig. 4F–I). To identify the role of PHTF2 in EMT of GC cells, EMT related proteins were examined by western blotting. The results implied that silencing *PHTF2* elevated E-cadherin and reduced vimentin expression in GC cells (Fig. 4C–E). Meanwhile, pcDNA3.1-*PHTF2*(p-*PHTF2*) was transfected into SGC-7901 and MGC-803 cells. RT-qPCR and western blotting analysis were testified the notable increaseof PHTF2 mRNA and protein levels (Fig. 5A–C). Furthermore, the results of wound healing assay and transwell assay exhibited that overexpression of PHTF2 dramatically promoted cell migration (Fig. 5F–I). In addition, the expression of E-cadherin observably declined but vimentin rose (Fig. 5C–E). Meanwhile, when PHTF2 was overexpressed in miR-30a-5p-overexpressing cells, the migratory and EMT-inhibitory phenotypes are reversed (Fig. S2). In conclusion, these results proved that PHTF2 positively regulated the migration and EMT of GC.

### miR-30a-5p suppressed GC cell migration and EMT by activating the AKT pathway

**Fig. 6:**
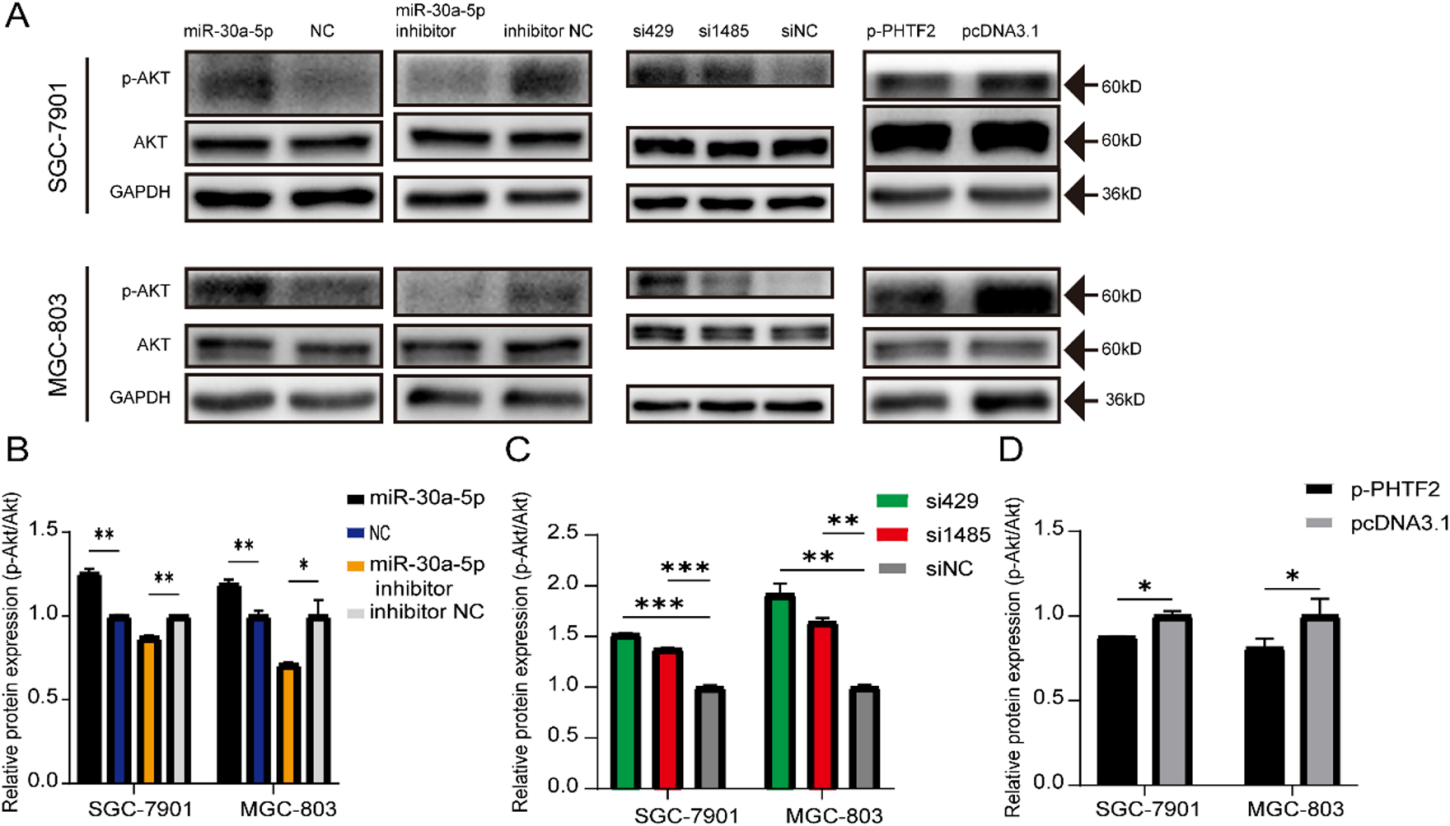
miR-30a-5p activated the PI3K/AKT pathway via targetting PHTF2. (**A**) The expression of p-AKT and AKT were measured by western blot. (**B**–**D**) Bar plots show the quantitative results of western blot. *p < 0.05, **p < 0.01, ***p < 0.001. *NC* negative control, *inNC* inhibitor negative control. The data expressed as the mean ± SD.

To more fully survey the regulatory pathway of miR-30a-5p/PHTF2 axis, we performed western blotting to detect the protein expression levels of key components in the AKT signalling pathway. The results showed that miR-30a-5p significantly increased p-AKT/AKT levels but miR-30a-5p inhibitor attenuated them in GC cells (Fig. 6A,B). Moreover, si429 and si1485 also enhanced p-AKT/AKT levels, while p-PHTF2 resulted in decreases in these levels in SGC-7901 and MGC-803 cells (Fig. 6A,C,D). In short, miR-30a-5p/PHTF2 may impede the EMT process and activate the AKT signalling pathway to restrain cell migration.

## Discussion

In our study, we first identified that miR-30a-5p dramatically inhibited the mRNA and protein of PHTF2 by its 3’UTR region binding sequence in GC cells. In addition, miR-30a-5p was downregulated in GC cells compared with GES-1. The wound healing assay and transwell assay results showed that miR-30a-5p impeded cell migration in MGC-803 and SGC-7901. Similarly, miR-30a-5p hampered cell migration in esophageal cancer^19^. Moreover, miR-30a-5p suppressed cell migration in MCF-7 and MDA-MB-453 cells^20^. Li et al.^21^. also reported that miR-30a-5p hindered migration by targetting LDHA in MDA-MB-231. As well, Meng et al.^22^. demonstrated that miR-30a-5p restrained cell migration in Non-small cell lung cancer cell lines.

To comprehensively clarify the potential molecular roles of miR-30a-5p in GC, we predicted putative target genes of miR-30a-5p in GC cells by bioinformatics analysis. Among the candidate target genes, we focused on *PHTF2. PHTF2*(putative homeodomain transcriptional factor2) located at 7q11.23-q21 of the human genome and was an evolutionarily conserved gene that mainly expressed in muscle tissue, which was related to the mediation of stress transcription^23^. Moreover, Hazra et al.^24^. indicated that PHTF2 was found as potential markers in recurrent implantation failure and pre-eclampsia. Chen et al.^25^. also implied that knockdown of *PHTF2* significantly weakened cell migration in aggressive PC3 cells. Molecular mechanisms underlying the roles of PHTF2 in various cancer are poorly characterized. In our study, we verified that knockdown of *PHTF2* inhibited cell migration in SGC-7901 and MGC-803, while overexpression of PHTF2 promoted cell migration. In summary, the results demonstrated that the effects of miR-30a-5p in GC were mediated by targetting PHTF2.

Epithelial-mesenchymal transition (EMT) refers to the biological process, which epithelial cells are transformed into mesenchymal phenotype cells through a specific program^26,27^. Furthermore, the hallmark of EMT is the decreased expression of cell adhesion proteins (such as E-cadherin and cytokeratin), while the expression of mesenchymal-related molecules (such as N-cadherin and vimentin) increases^28^. Therefore, EMT plays a crucial role in cancer migration. In recent years, a large number of studies have confirmed that miRNAs were related to EMT in malignant tumors. In triple negative breast cancer, Noyan et al.^29^ suggested that miR-770-5p decreased invasion capacity by inhibiting EMT through targetting DMNT3A in TNBC. In addition, in ovarian cancer, Wang et al.^30^ illustrated that miR-149-3p promoted EMT by downregulating TIMP2 and CDKN1A. In this study, we proved that miR-30a-5p could inhibit EMT by observably improving expression levels of E-cadherin, while reducing the levels of vimentin. These findings corresponded to our migration results.

**Fig. 7:**
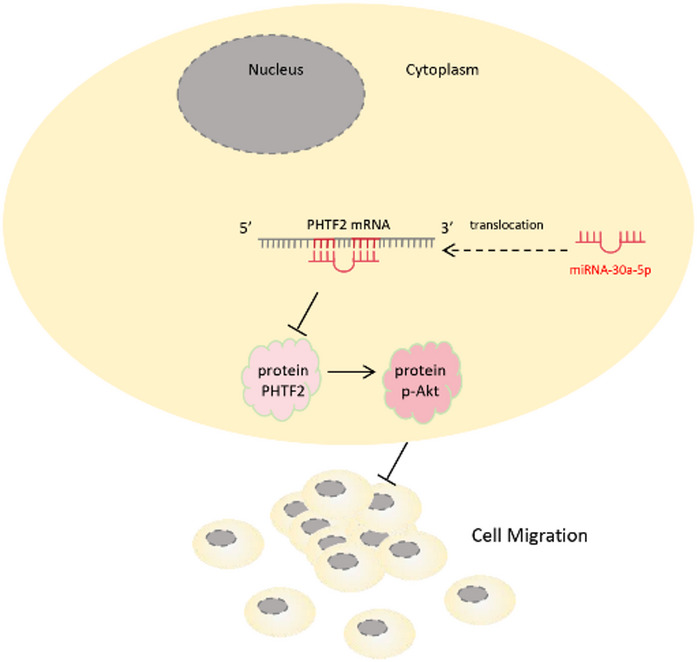
A schematic diagram illustrated that miR-30a-5p inhibited gastric cancer cell migration via the PHTF2/AKT axis.

Previous studies have implied that the AKT signaling pathway linked with tumor metastasis. For example, Xie et al.^31^ showed that miR-6875-3p promoted the invasion and metastasis of hepatocellular carcinoma via BTG2/FAK/AKT pathway. Similarly, Wang et al.^32^ confirmed that miR-29b inhibited EMT and suppressed phosphorylation of AKT. Therefore, we researched the AKT signalling pathway in the roles of alterations in miR-30a-5p expression in GC cells. Our data indicated that miR-30a-5p activated the AKT signalling pathway by downregulation of PHTF2 (Fig. 7).

However, the present study does have some limitations, such as incomplete validation of the AKT signalling pathway, role of p-AKT in cell migration is not clearly explained and localization of p-AKT to the cellular membrane regulating migration needs to be clarified. While PHTF2 over-expression or knock-down has clear phenotypic effects, this study does not explain how PHTF2 signals to AKT. At least one direct line of evidence is required—for example, a phospho-AKT western blot with densitometric analysis following PHTF2 manipulation, or a co-IP/ChIP experiment showing interaction between PHTF2 and AKT. Therefore, we will perform additional loss-of-function experiments to explain the role of p-AKT in cell migration in the future. Meanwhile, it is necessary to confirm the relationship between miR-30a-5p-PHTF2 and AKT pathway with CRISPR knockout of PHTF2 in a larger population.

In conclusion, our findings identified that miR-30a-5p was down-regulated in GC cells. Moreover, miR-30a-5p inhibited GC cell migration and EMT. Meanwhile, miR-30a-5p suppressed PHTF2 expression by directly binding to the *PHTF2* 3’UTR. Finally, we confirmed that AKT signalling pathway played a vital role in this process. Hence, these data further supported that PHTF2 promoted migration and EMT progression in GC by regulating AKT signalling pathway, implicating PHTF2 as a valuable predictor of prognosis in GC and a potential therapeutic target for GC.

## Conclusions

In summary, we confirmed for the first time that *PHTF2* was a direct target gene of miR-30a-5p in GC cells. In addition, miR-30a-5p inhibited the migration and EMT of GC cells by targetting PHTF2 via activating the AKT signalling pathway. Our results suggested that the miR-30a-5p/PHTF2/AKT axis might serve as a possible therapeutic target for GC patients.

## Supplementary Information

Below is the link to the electronic supplementary material.

Supplementary Material 1

Supplementary Material 2

Supplementary Material 3

Supplementary Material 4

Supplementary Material 5

Supplementary Material 6

Supplementary Material 7

Supplementary Material 8

## Data Availability

The data will be available on reasonable request. If someone wants to request the data from this study, please contact Fei Tu.
