# Correction: MiR-30a-5p activates the AKT signalling pathway by targeting PHTF2 to inhibit migration and EMT of gastric cancer

**DOI:** 10.1038/s41598-026-54657-z

**Published:** 2026-05-28

**Authors:** Fei Tu, Fengyuan He, Zhiyuan Li, Yiqing Jia, Lingzhu Wang, Tiesuo Zhao, Sheng Guo, Yan Jin, Zhijun Yang

**Affiliations:** 1 School of Forensic Medicine, Xinxiang Medical University, Xinxiang, China; 2 Xinxiang Engineering Technology Research Center of Immune Checkpoint Drug for Liver-Intestinal Tumors, Xinxiang Medical University, Xinxiang, China; 3 School of Basic Medical Sciences, Xinxiang Medical University, Xinxiang, China

Correction to: *Scientific Reports* 10.1038/s41598-025-33375-y, published online 20 December 2025

The original version of this Article contained errors in Figure 2. Specifically in panel B, the image representing MGC-803 cells in the NC (negative control) group was erroneously replaced with another image. The original Figure 2 appears below.

The original Article has been corrected.

**Figure 2:**
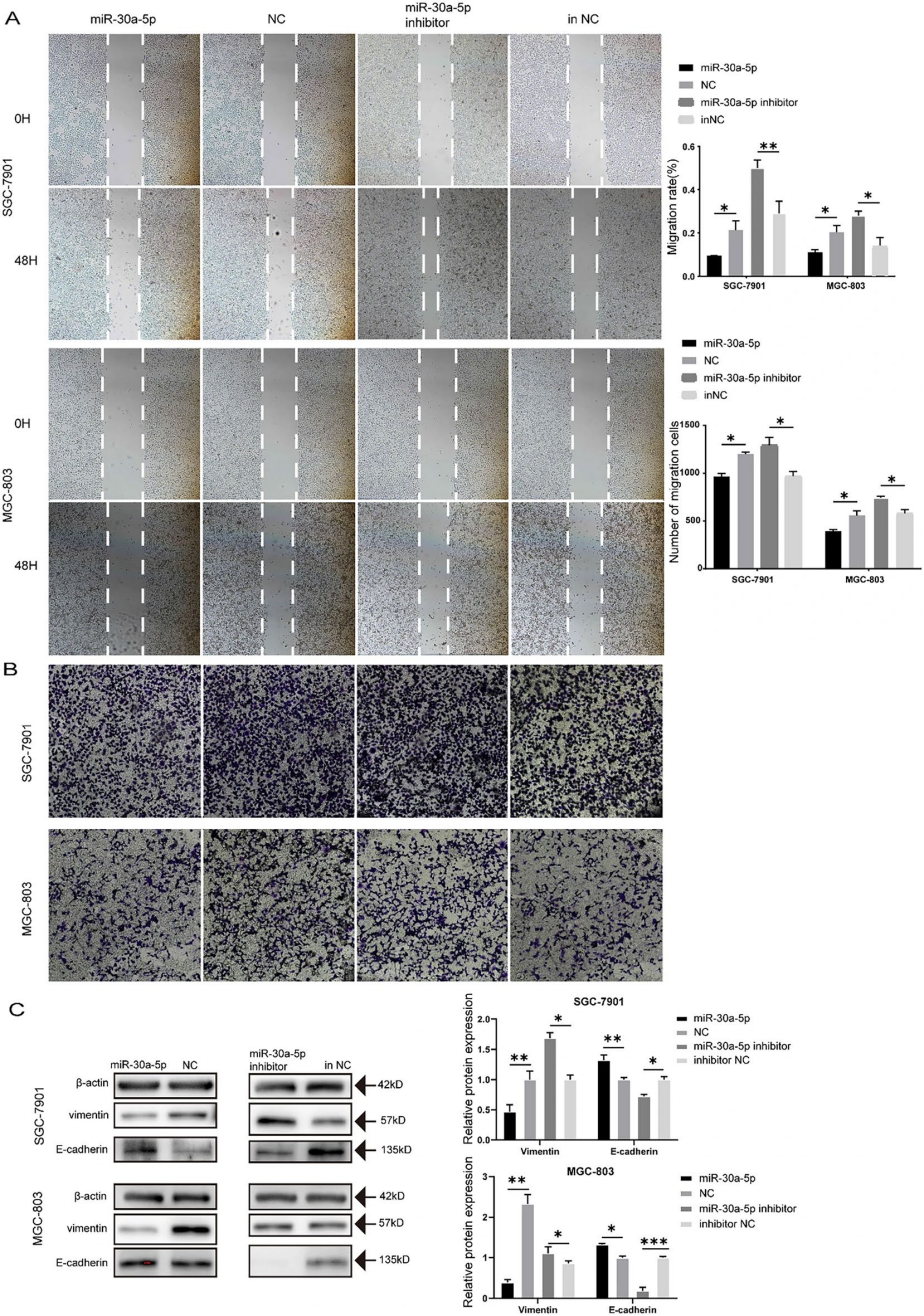
miR-30a-5p inhibited gastric cancer cell migration and Epithelial-Mesenchymal Transition (EMT). (**A**) The wound healing assays were performed to assess the effect of miR-30a-5p on cell motility at 0 and 48 H. (**B**) The transwell assays were performed to detect the effect of miR-30a-5p on migration. (**C**) The effect of miR-30a-5p on E-cadherin and vimentin protein expression in MGC-803 and SGC-7901 cells was detected by Western blot. *p < 0.05, **p < 0.01, ***p < 0.001. *NC* negative control, *inNC* inhibitor negative control. The data expressed as the mean ± SD.

