# Supplementary material for: MiR-30a-5p activates the AKT signalling pathway by targeting PHTF2 to inhibit migration and EMT of gastric cancer
Source: Sci Rep. 2025 Dec 20;16:3401. doi: 10.1038/s41598-025-33375-y (PMC12835005; doi:10.1038/s41598-025-33375-y)

**Fig.2** 7901 30a NC

β-actin


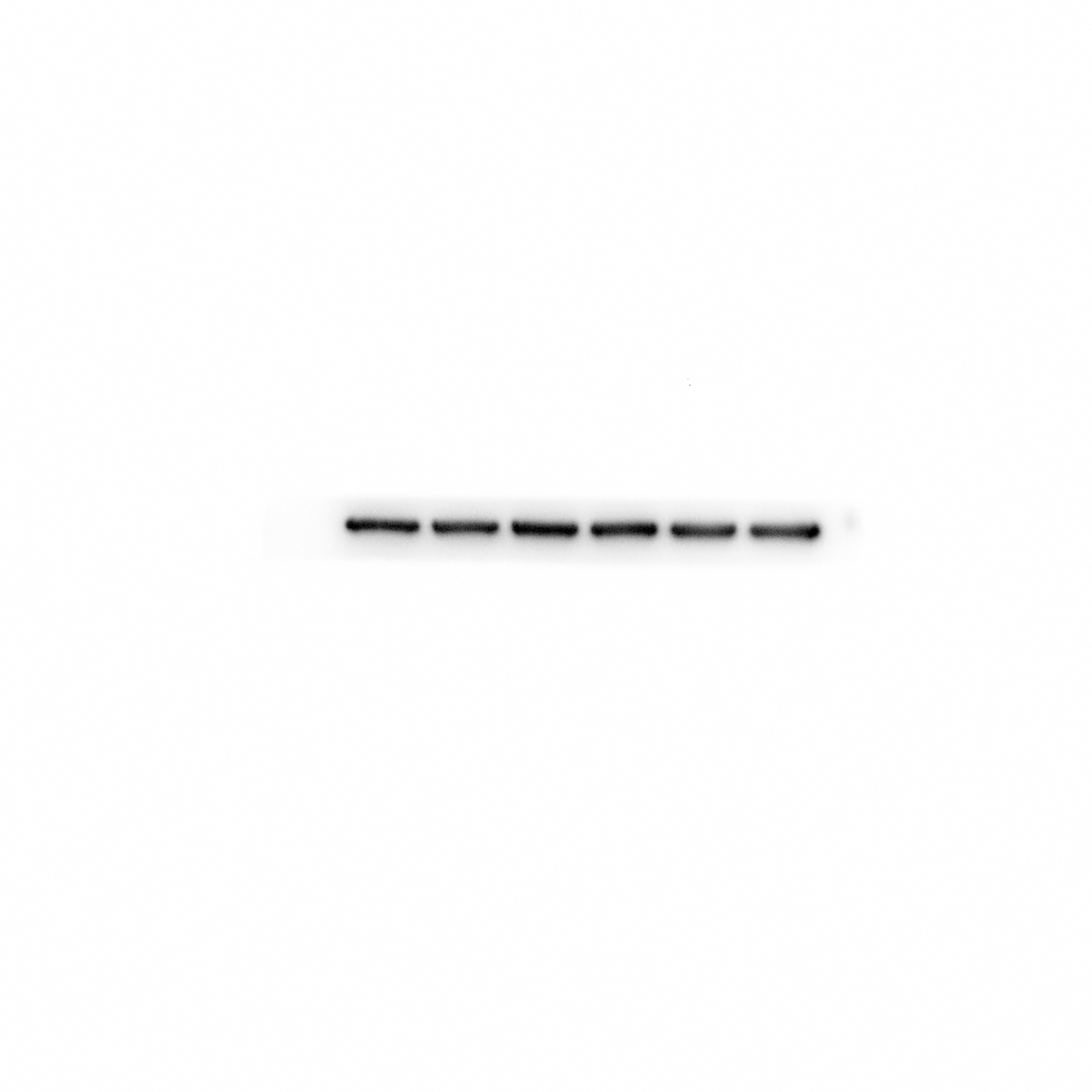


Vimentin


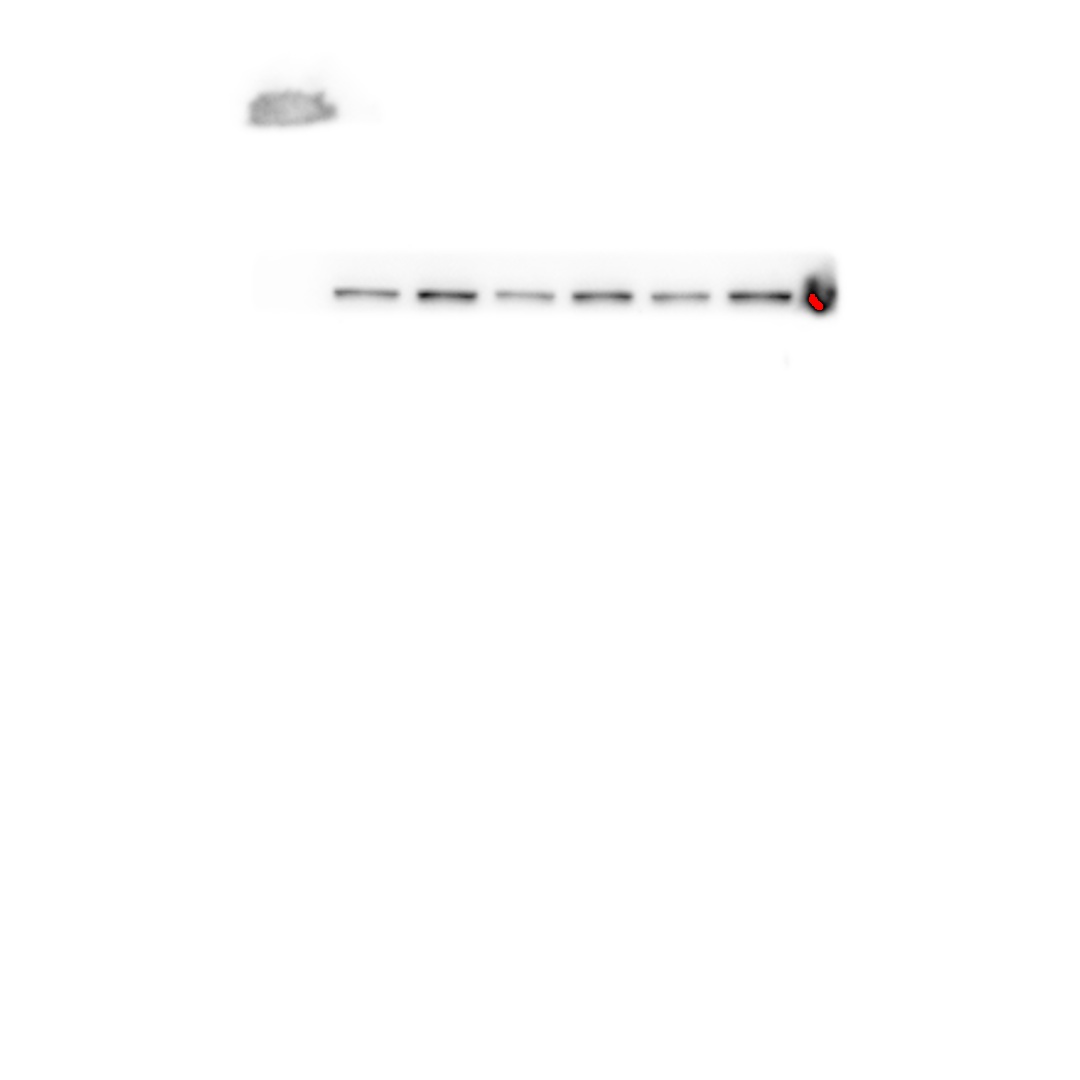


E-cadherin


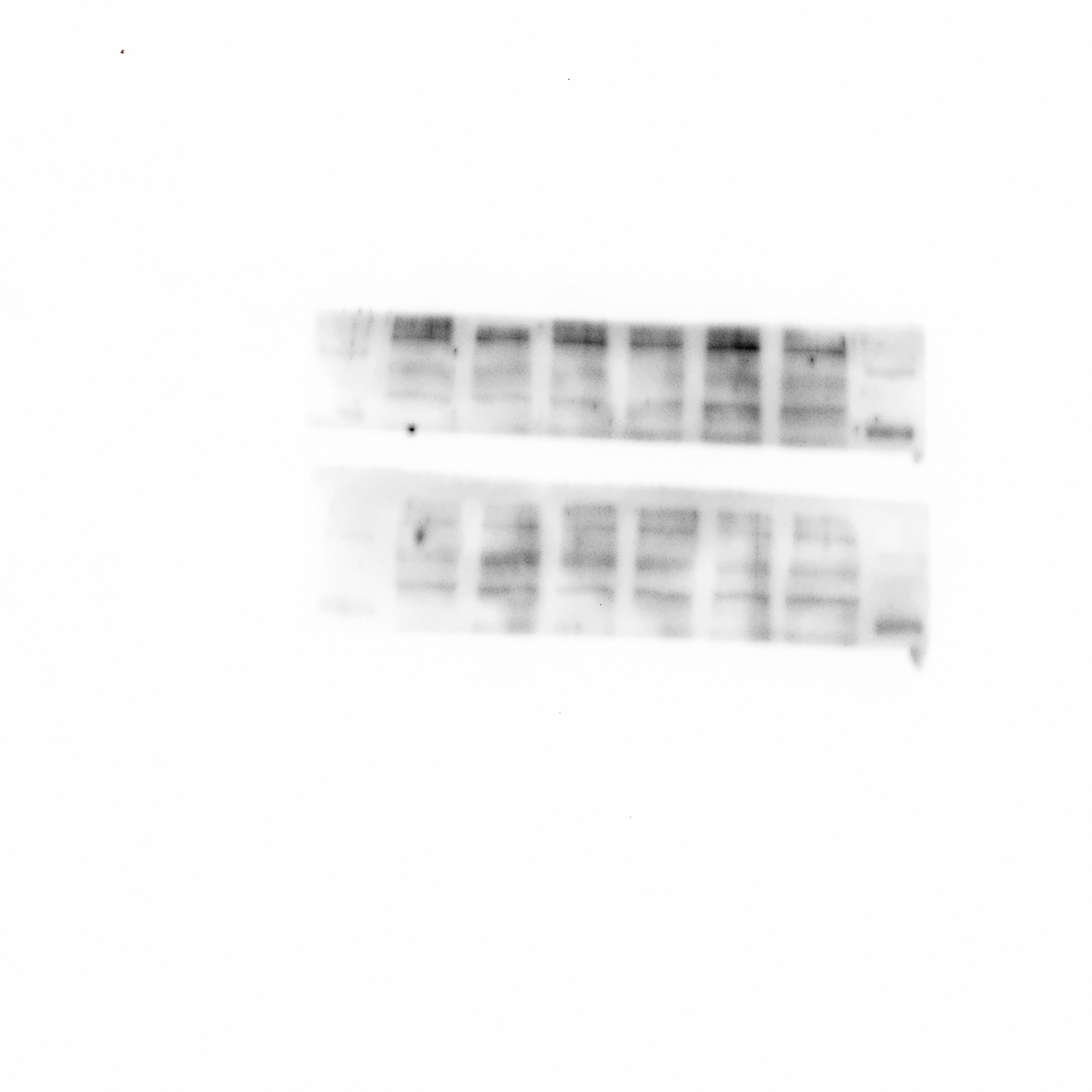


**Fig.2** 7901 30a in inNC

β-actin

**

**

Vimentin





1. cadherin


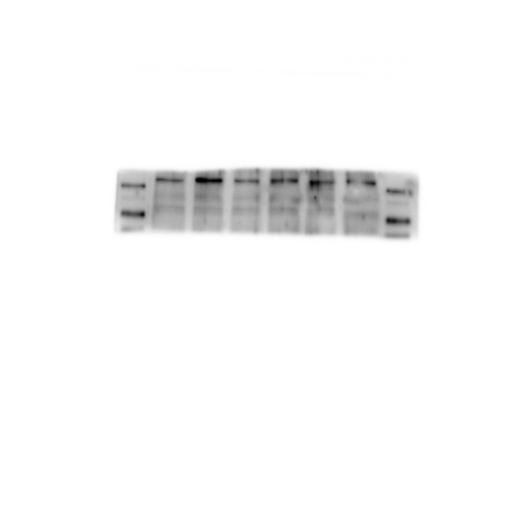


**Fig.2** 803 30a NC

β-actin


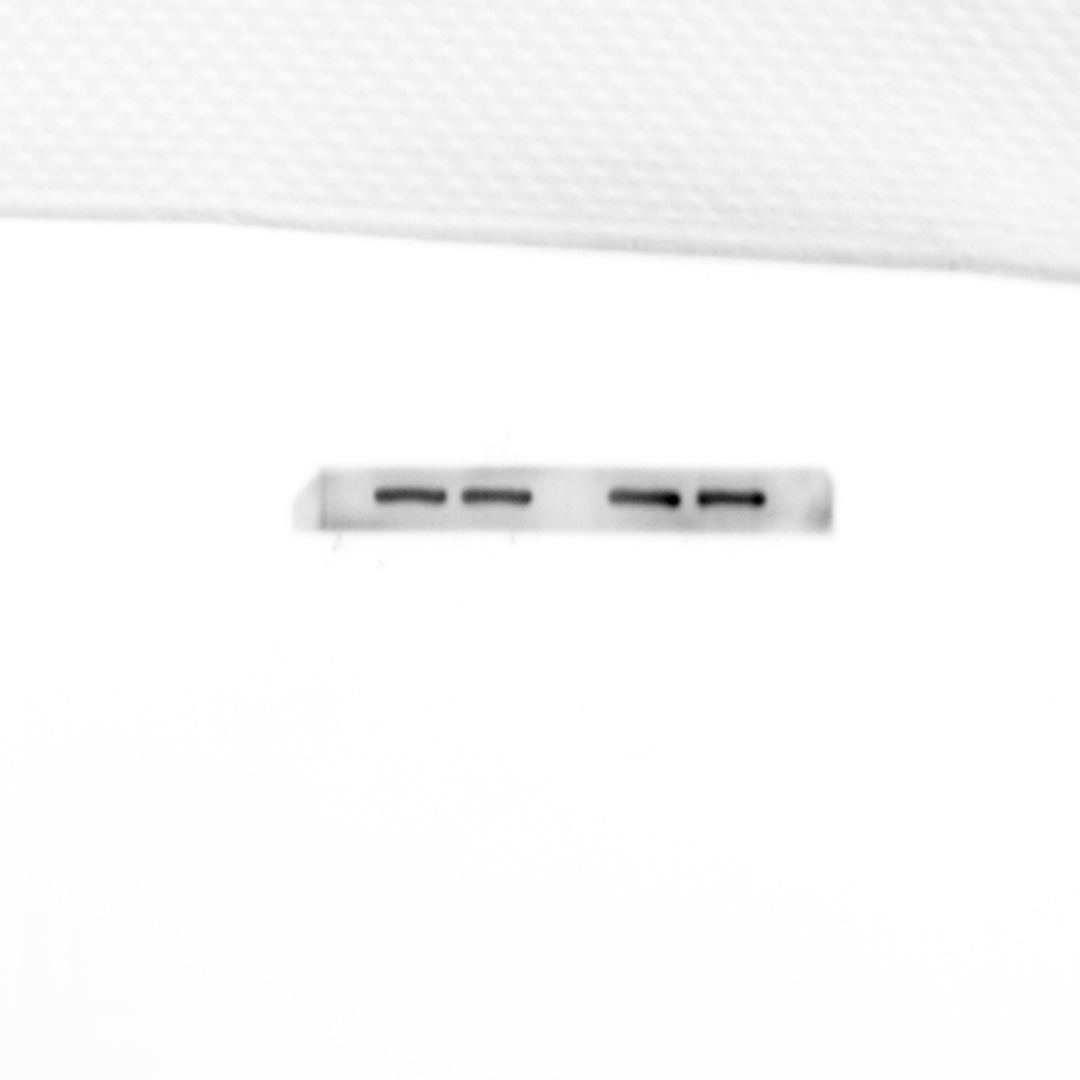


Vimentin


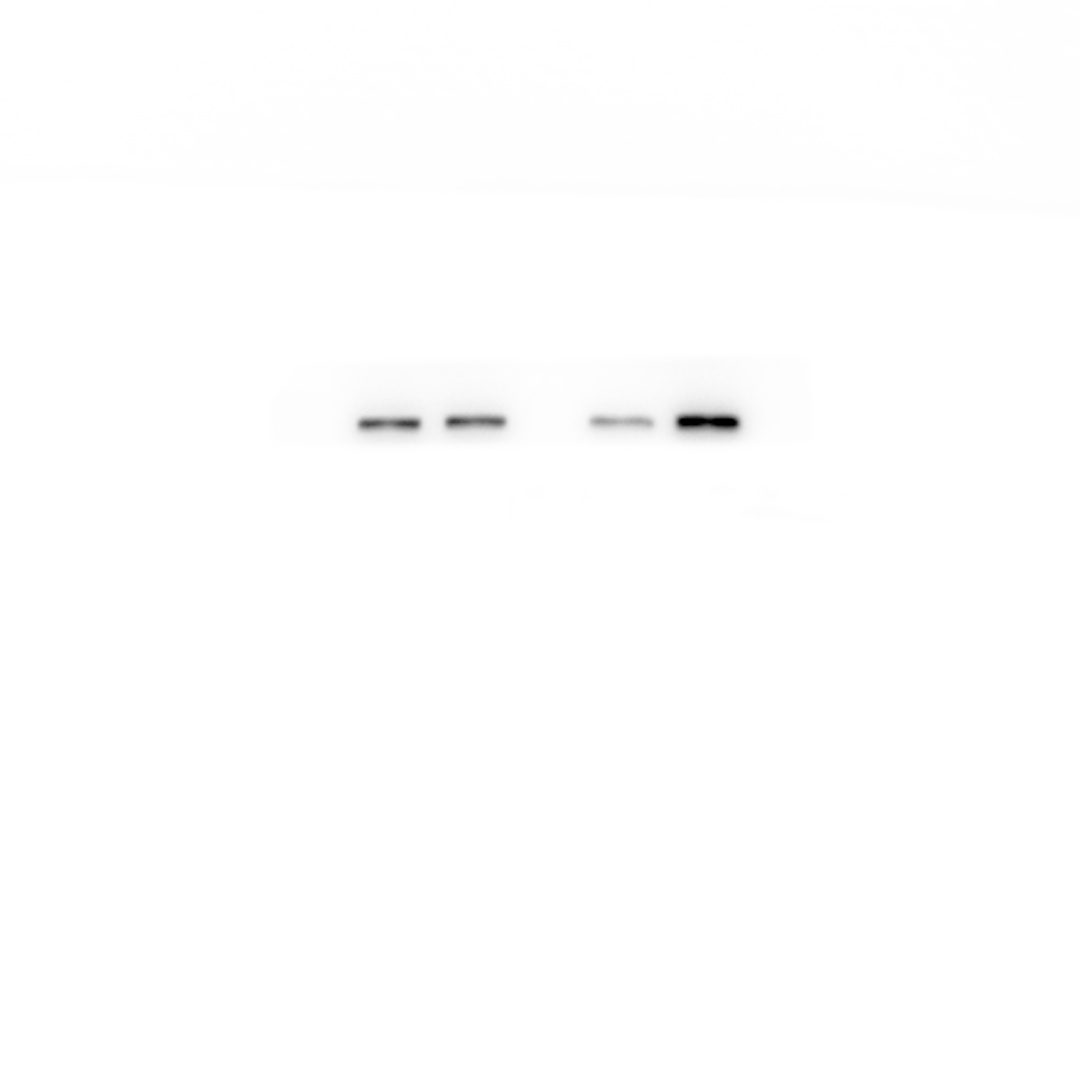


E-cadherin


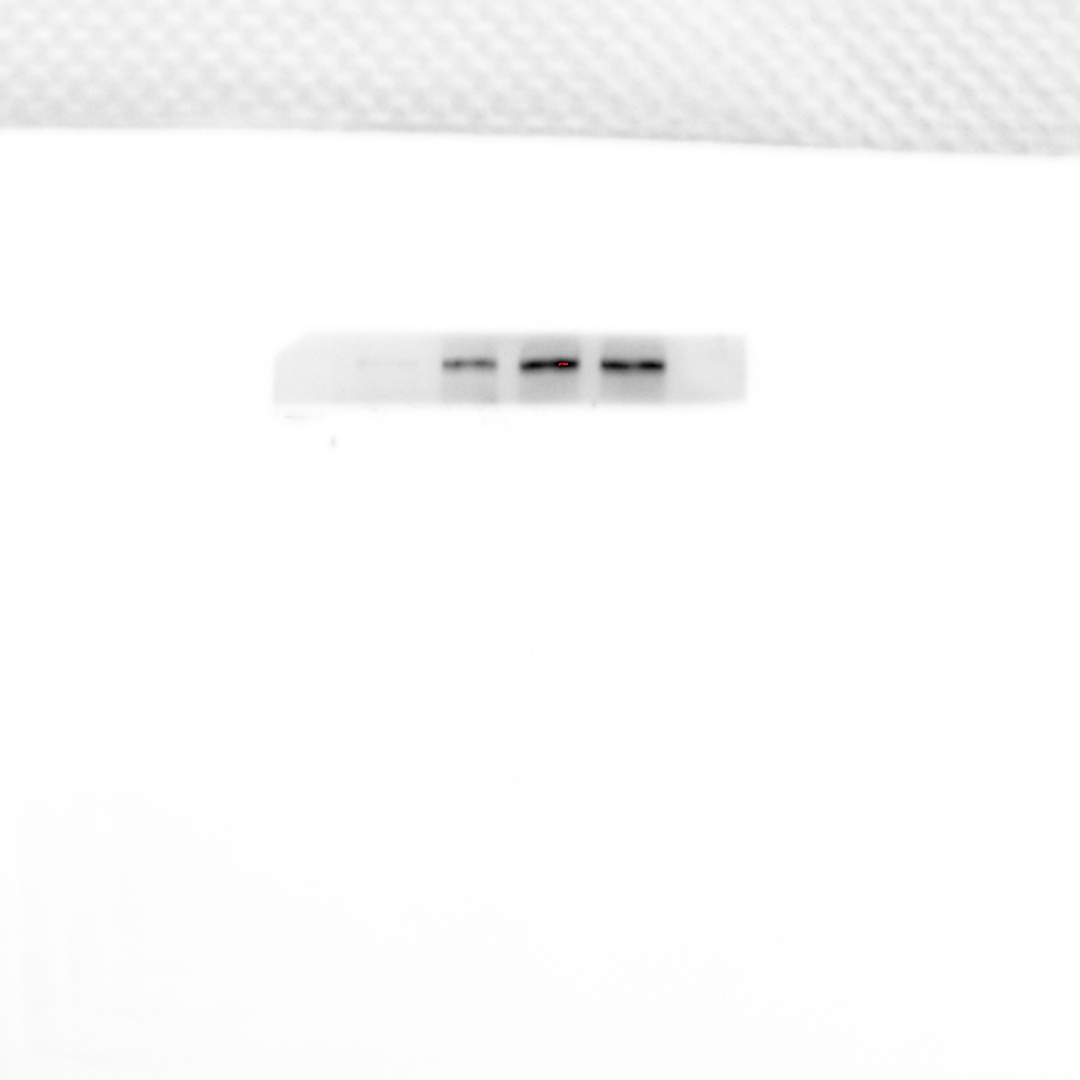


**Fig.2** 803 30a in inNC

β-actin


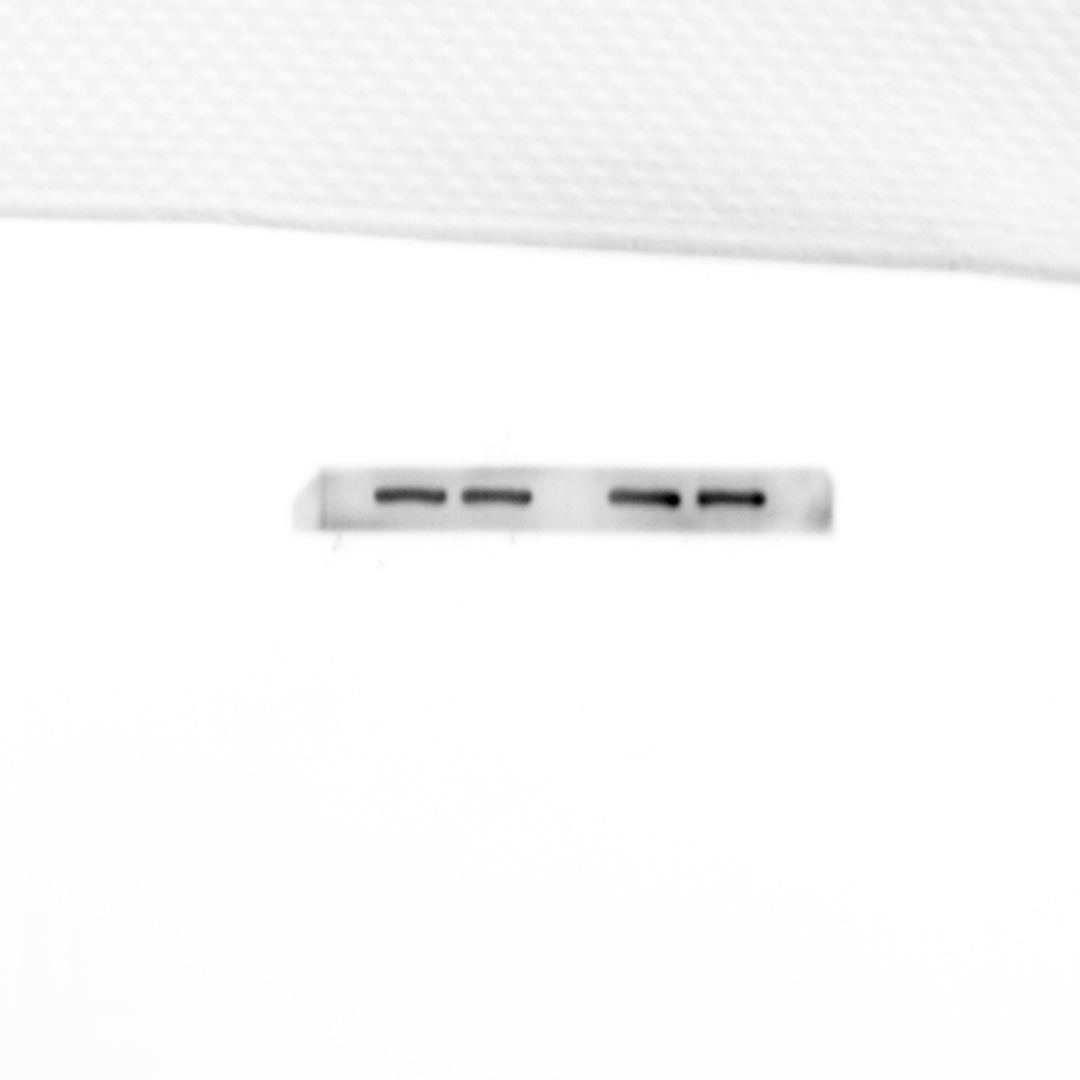


Vimentin


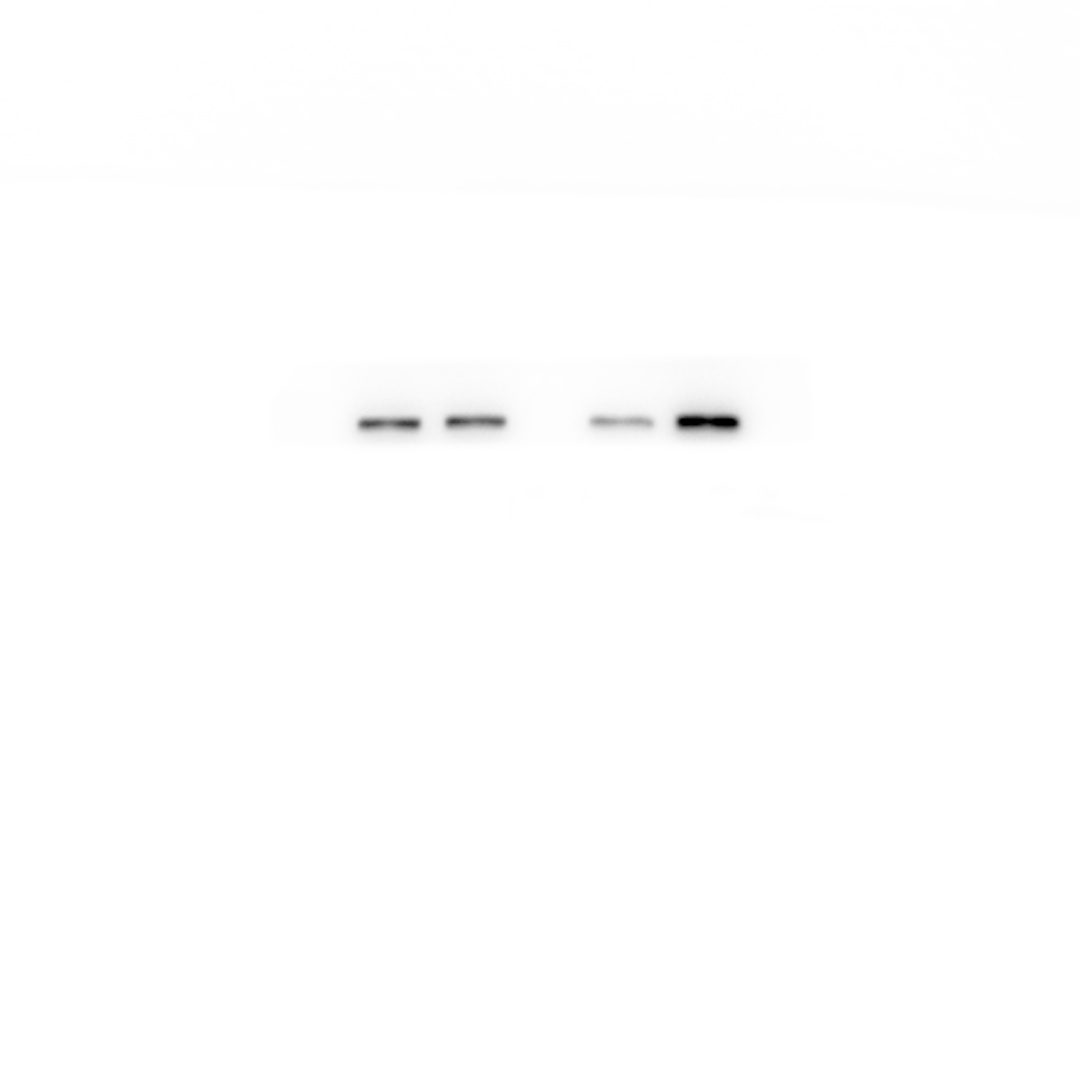


E-cadherin


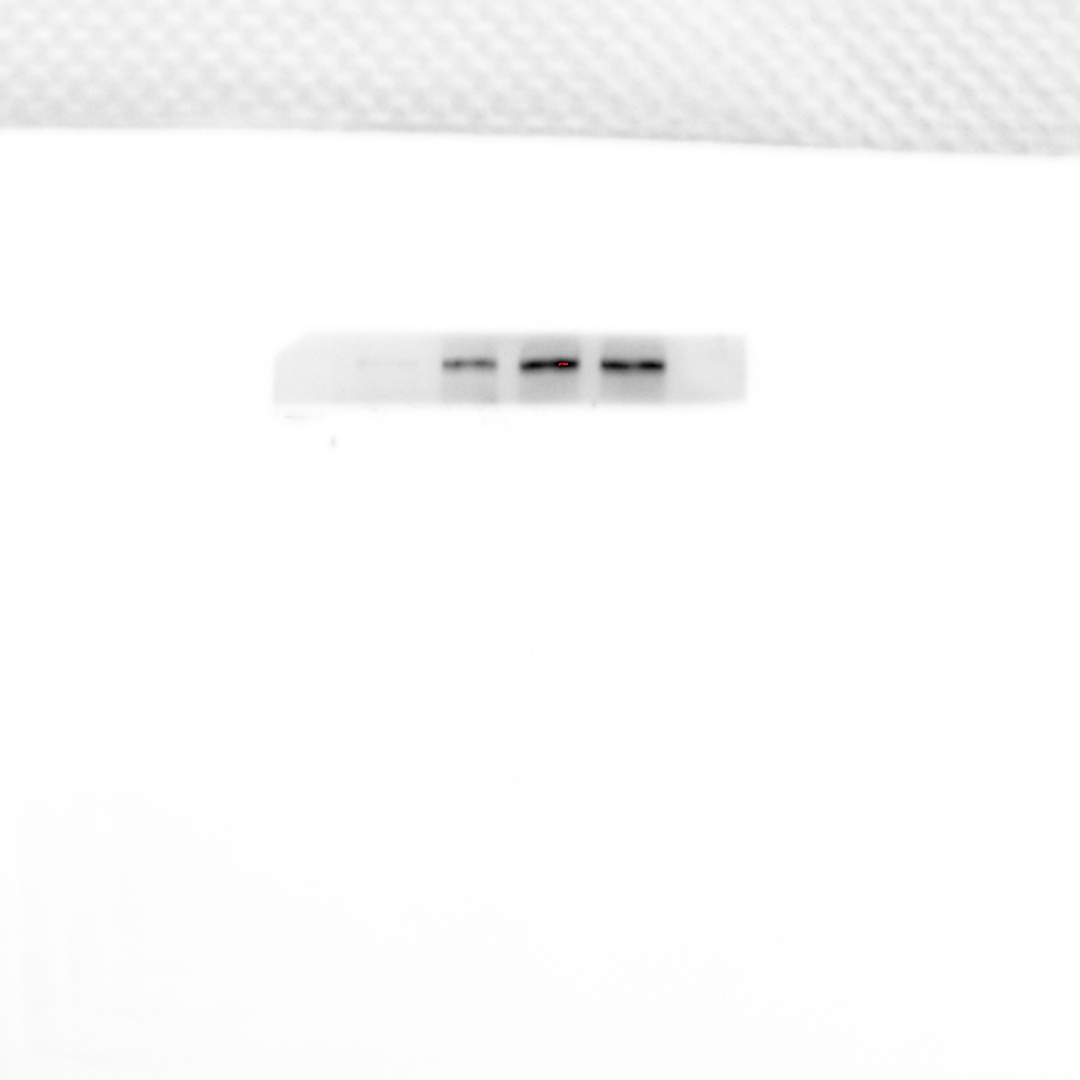


**Fig.2** 803 30a NC

β-actin ( E-cadherin )


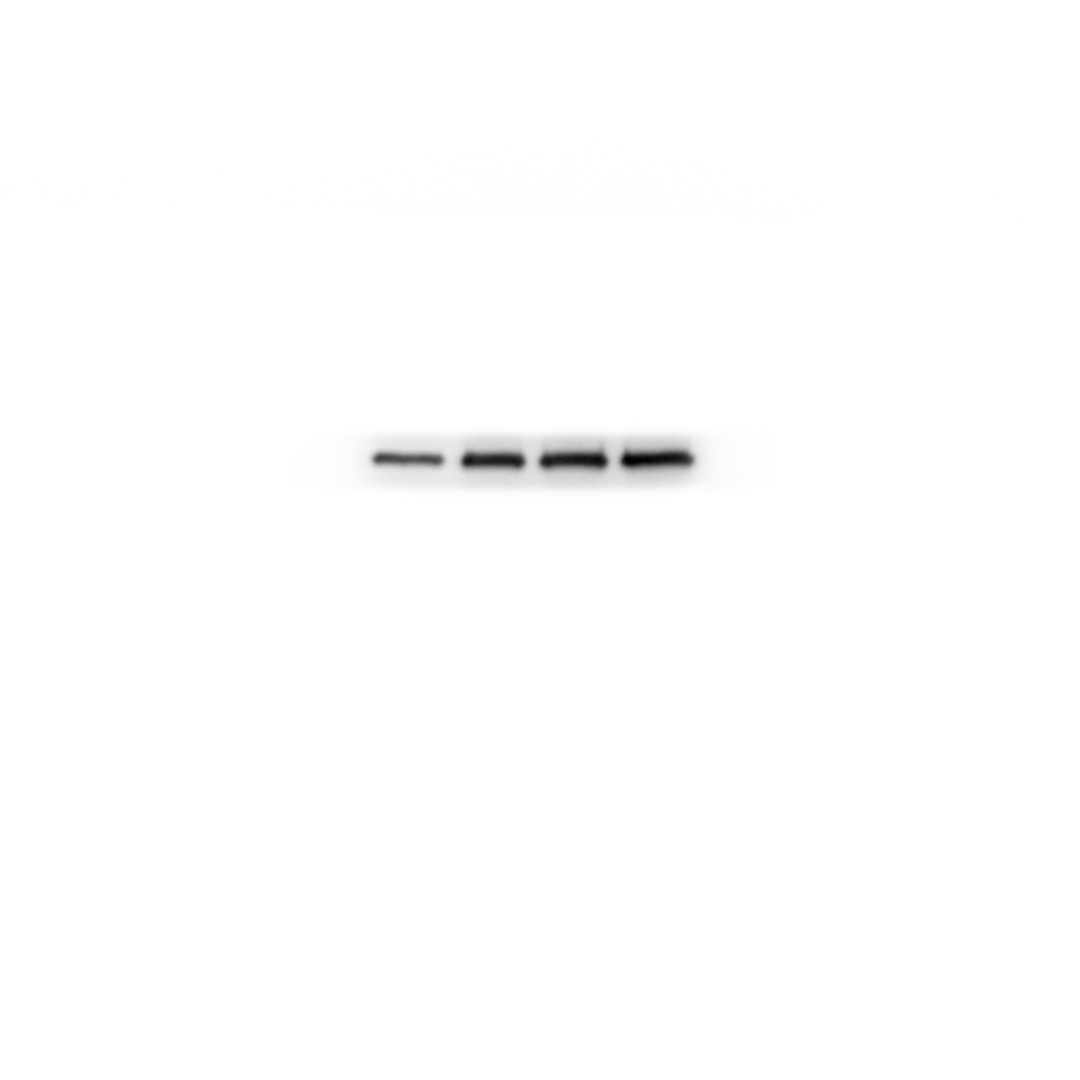


**Fig.2** 803 30a on inNC

β-actin ( E-cadherin )


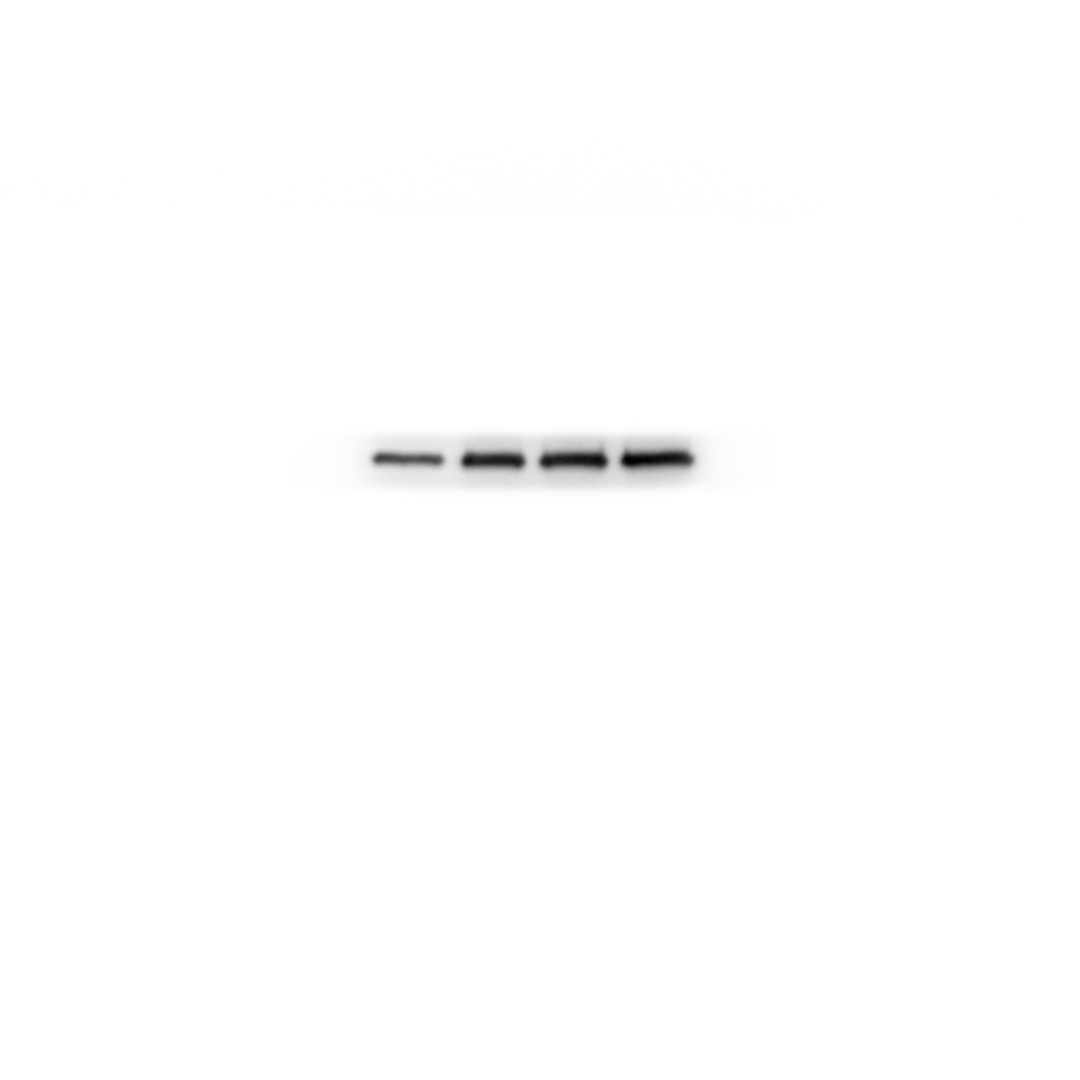

Supplement: Supplementary file 1 — Supplementary Material 1 [file 41598_2025_33375_MOESM1_ESM.docx]
