# Supplementary figures and images for: MiR-30a-5p activates the AKT signalling pathway by targeting PHTF2 to inhibit migration and EMT of gastric cancer
Source: Sci Rep. 2025 Dec 20;16:3401. doi: 10.1038/s41598-025-33375-y (PMC12835005; doi:10.1038/s41598-025-33375-y)

**Fig.3** 7901 30a NC

β-actin


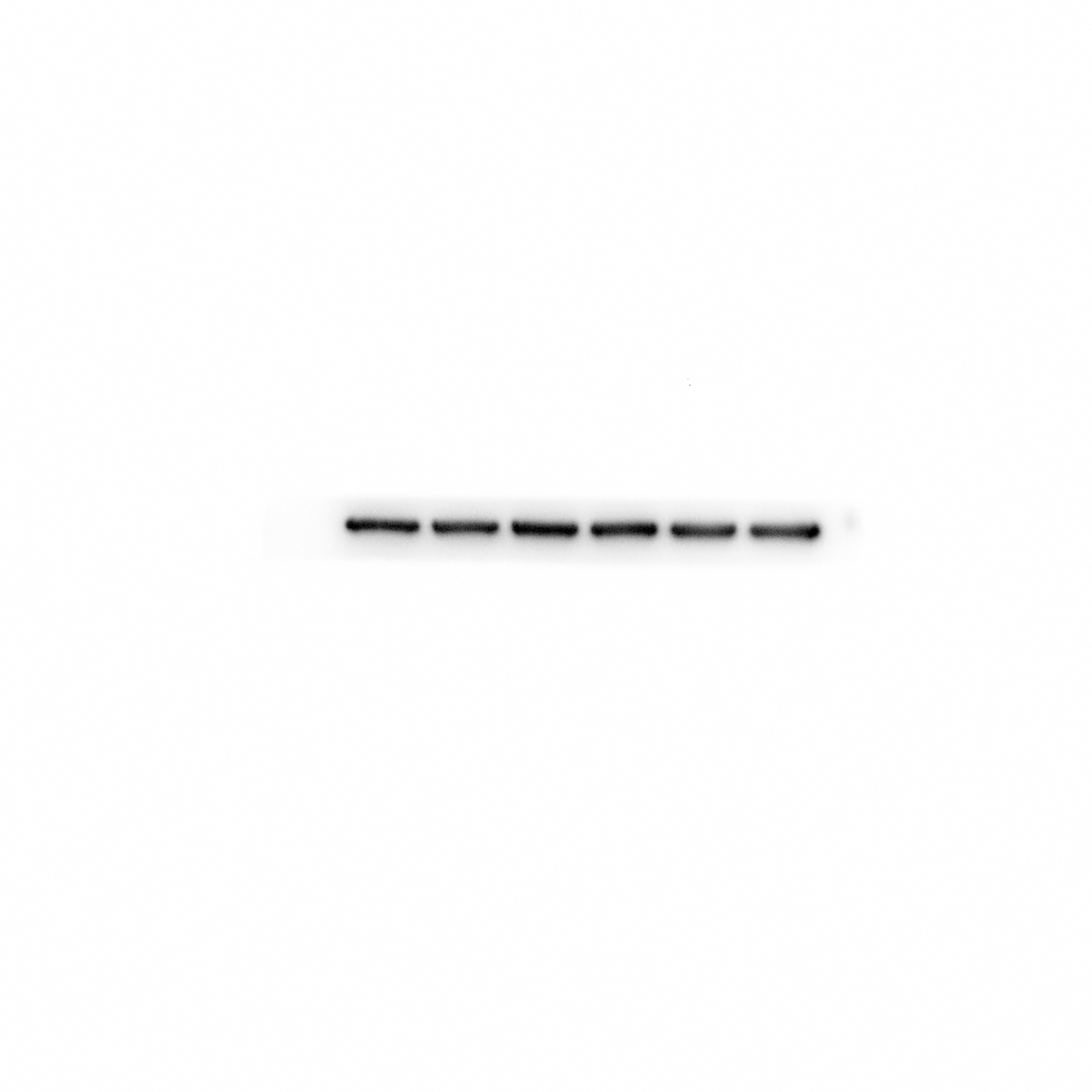


PHTF2


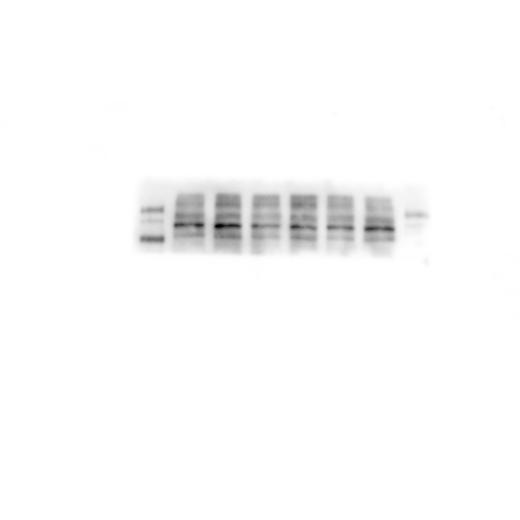


**Fig.3** 7901 30a in inNC

β-actin


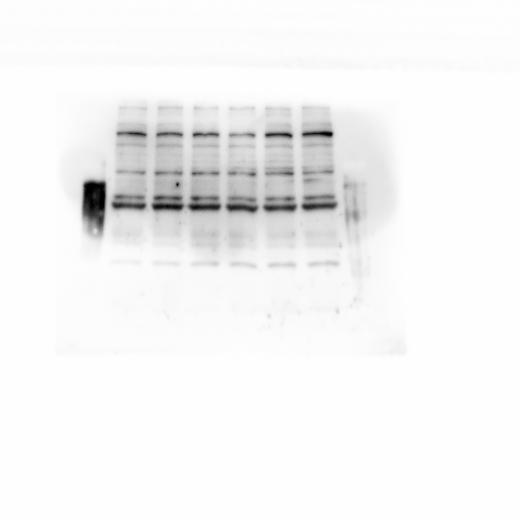


PHTF2


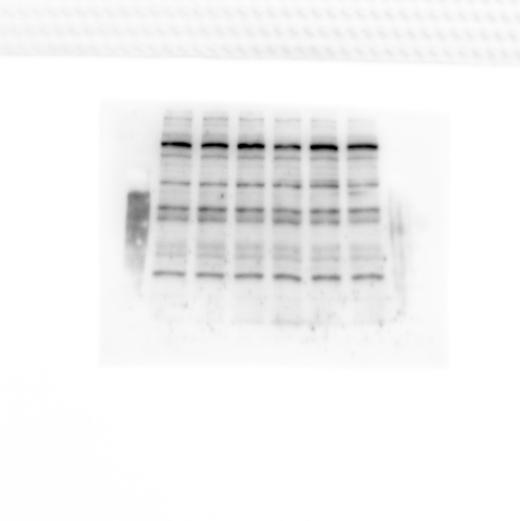


**Fig.3** 803 30a NC

β-actin


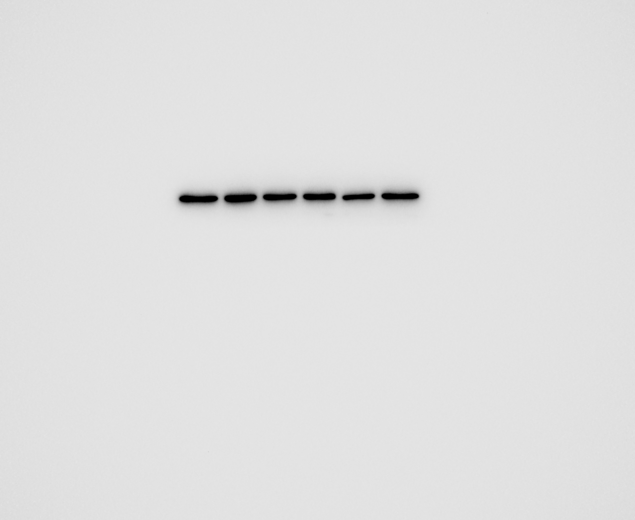


PHTF2


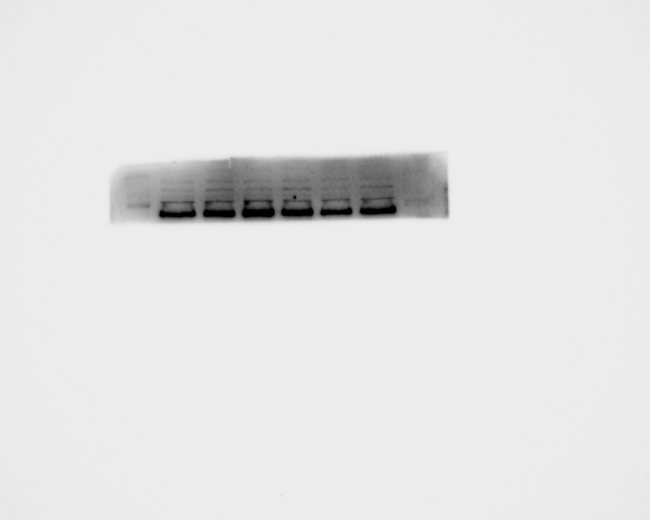


**Fig.3** 803 30a in inNC

β-actin


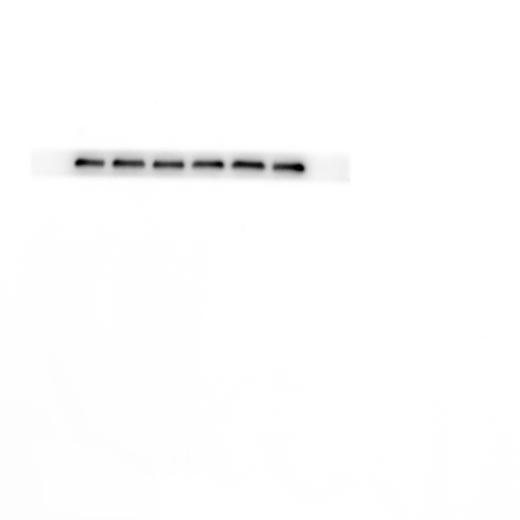


PHTF2


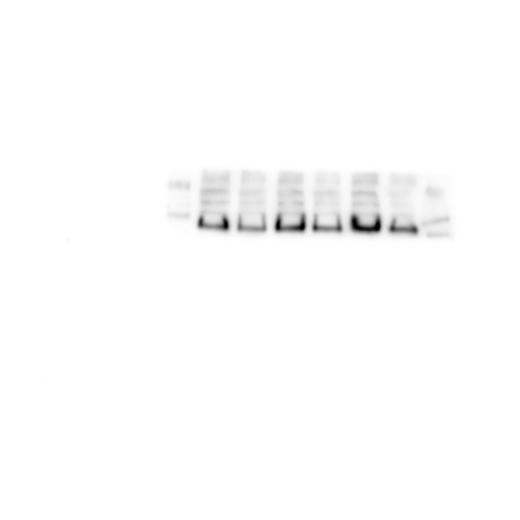

Supplement: Supplementary file 2 — Supplementary Material 2 [file 41598_2025_33375_MOESM2_ESM.docx]

**Fig.2** 7901 si429 si1485 siNC

β-actin


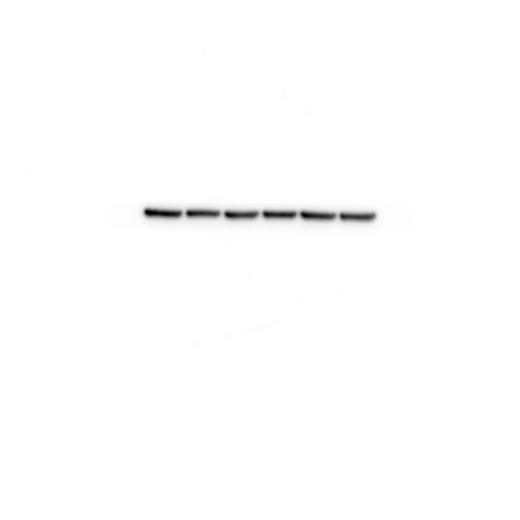


PHTF2


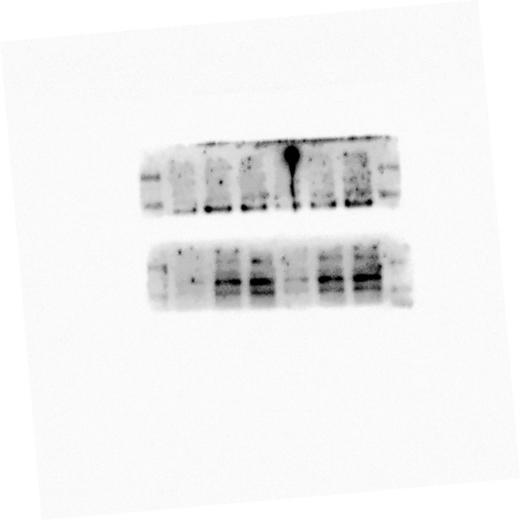


E-cadherin


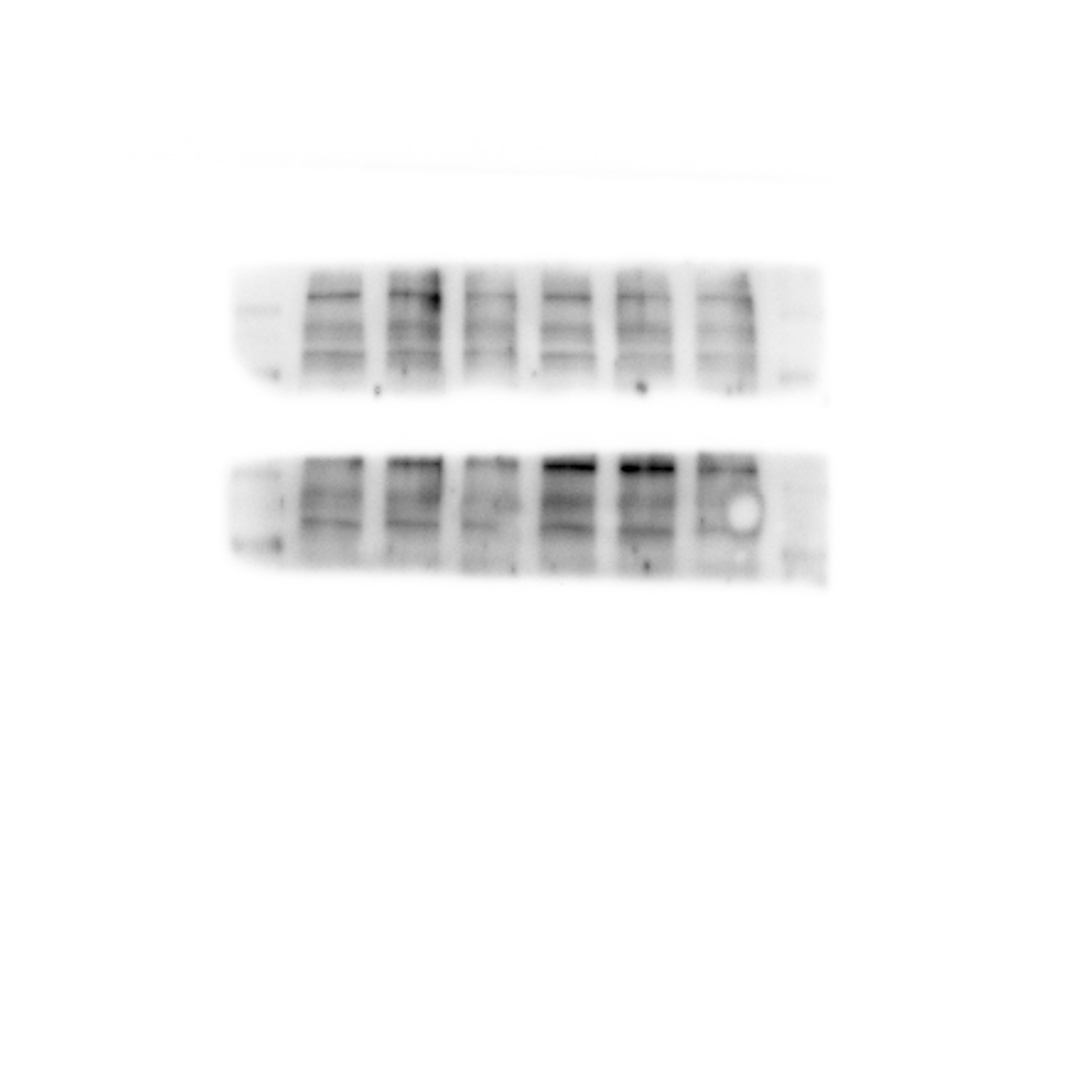


Vimentin


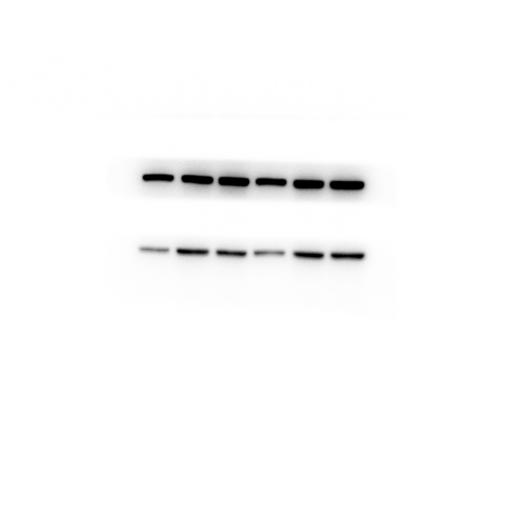


**Fig.2** 803 si429 si1485 siNC

β-actin


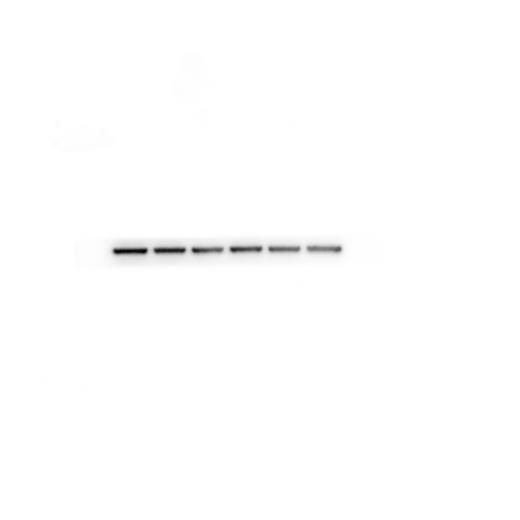


PHTF2


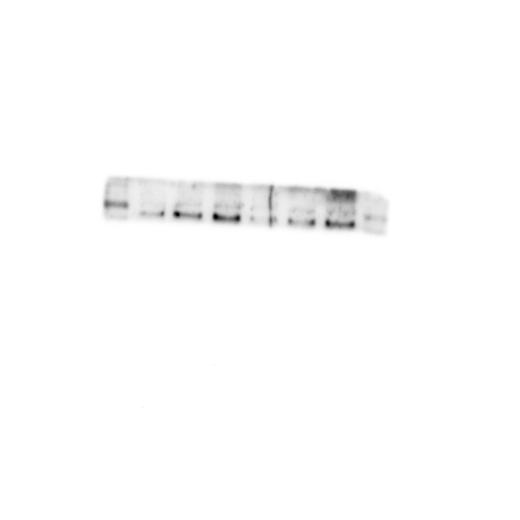


E-cadherin


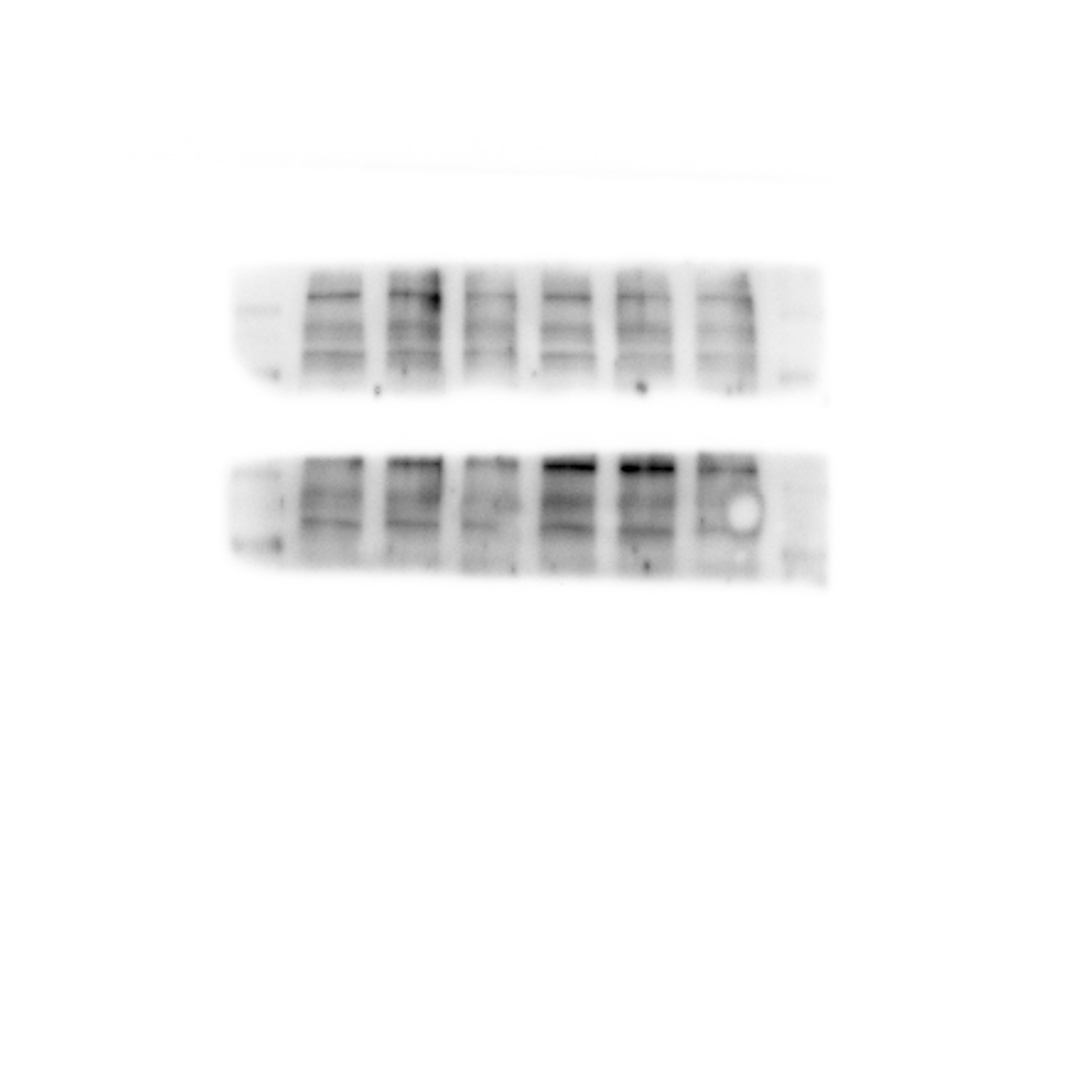


Vimentin


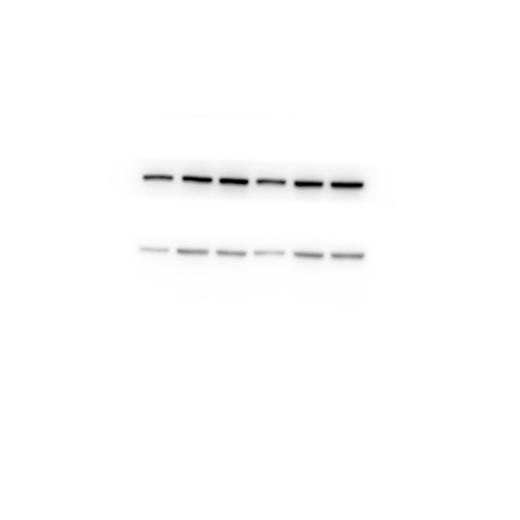

Supplement: Supplementary file 3 — Supplementary Material 3 [file 41598_2025_33375_MOESM3_ESM.docx]

**Fig.5** 7901 pcDNA3.1 p-PHTF2

GAPDH


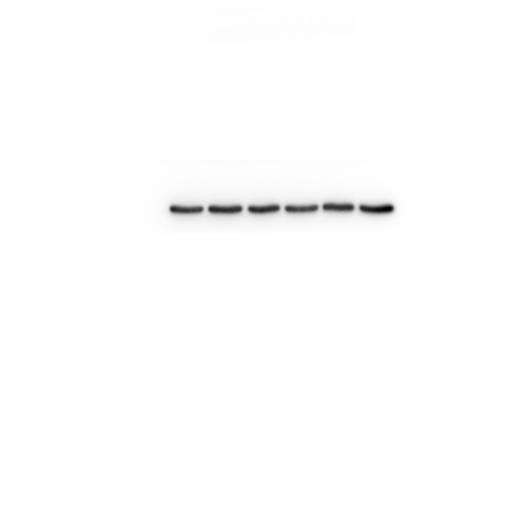


PHTF2


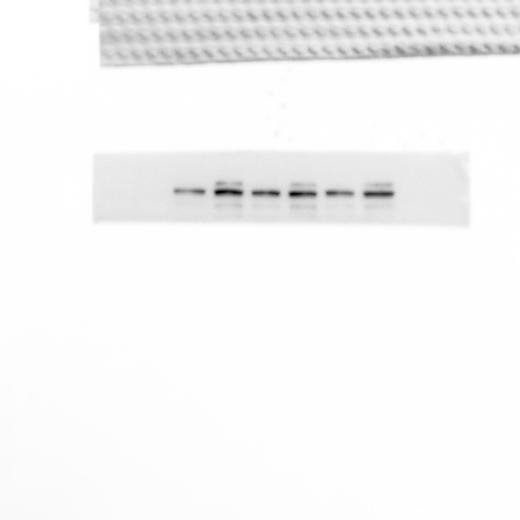


E-cadherin


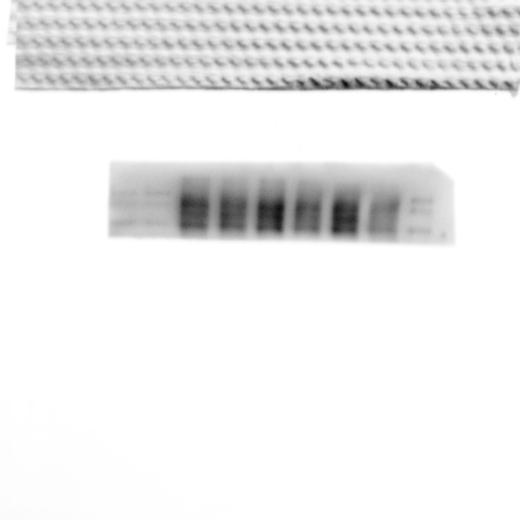


Vimentin


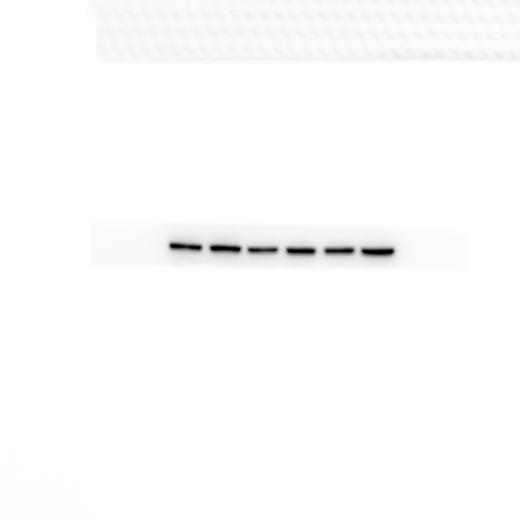


**Fig.5** 803 pcDNA3.1 p-PHTF2

GAPDH


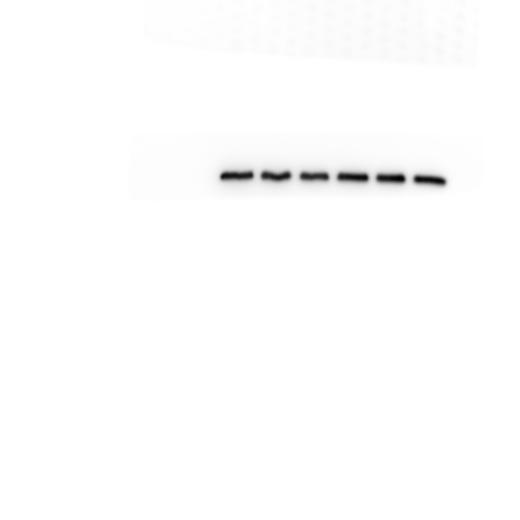


PHTF2


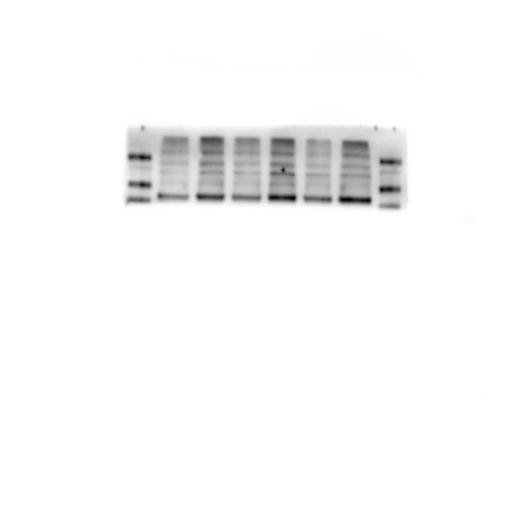


E-cadherin


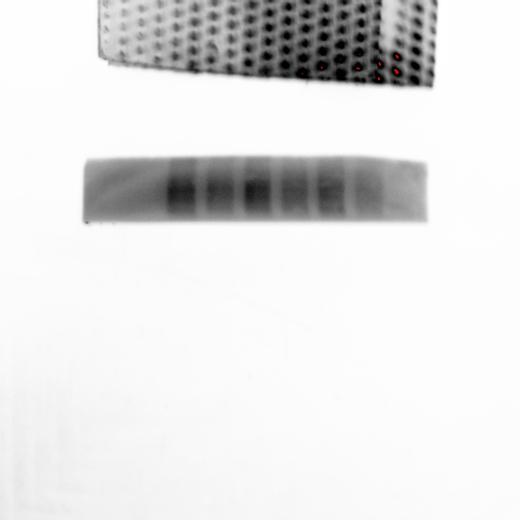


Vimentin


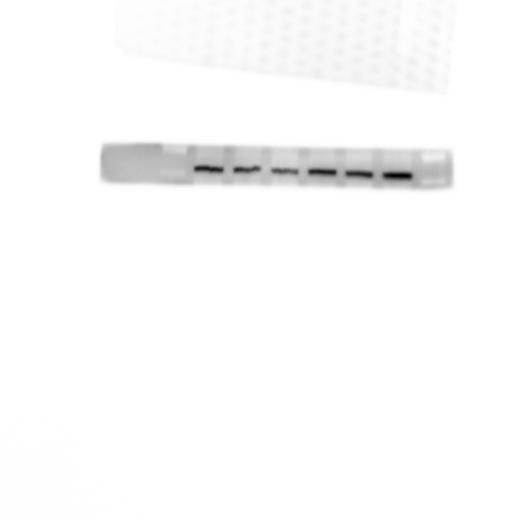

Supplement: Supplementary file 4 — Supplementary Material 4 [file 41598_2025_33375_MOESM4_ESM.docx]

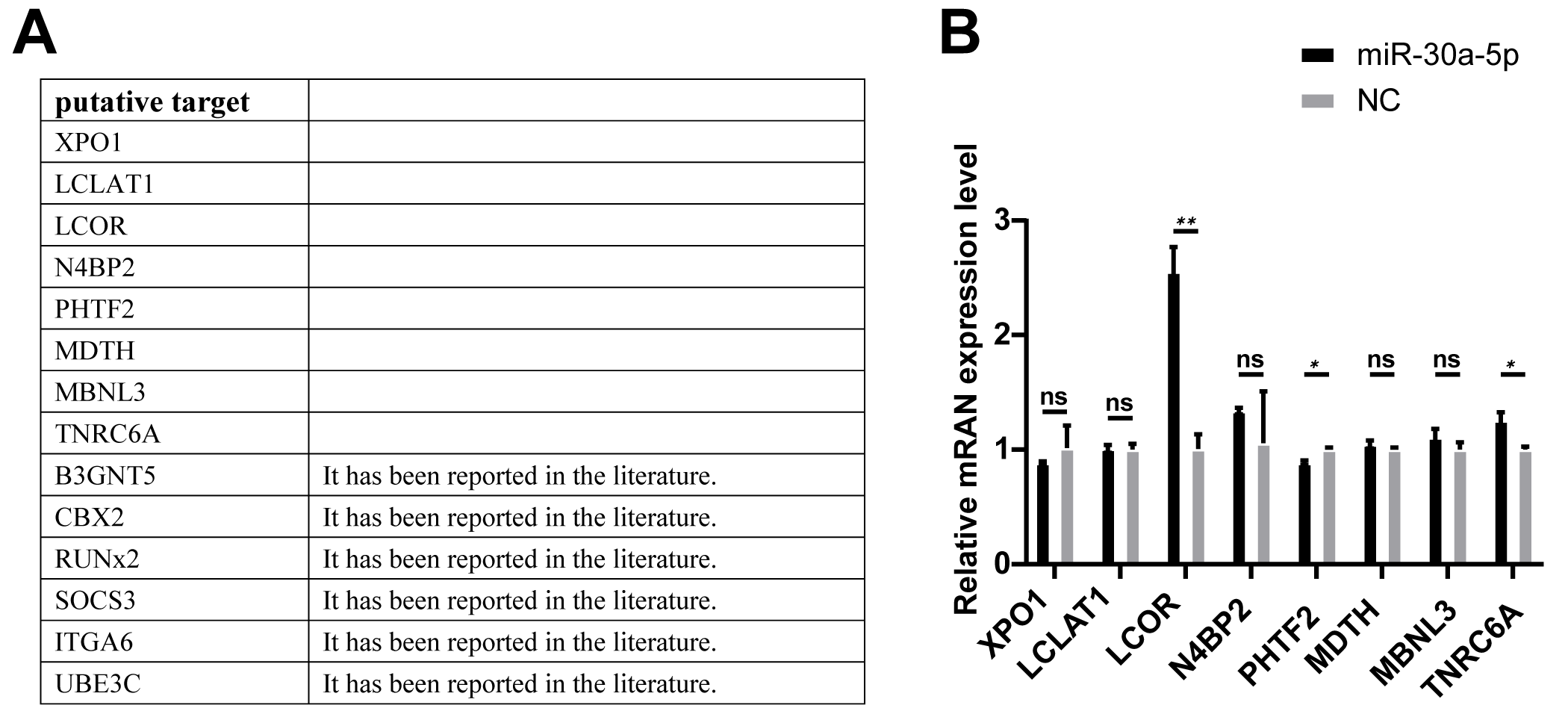

Supplement: Supplementary file 6 — Supplementary Material 6 [file 41598_2025_33375_MOESM6_ESM.tif]

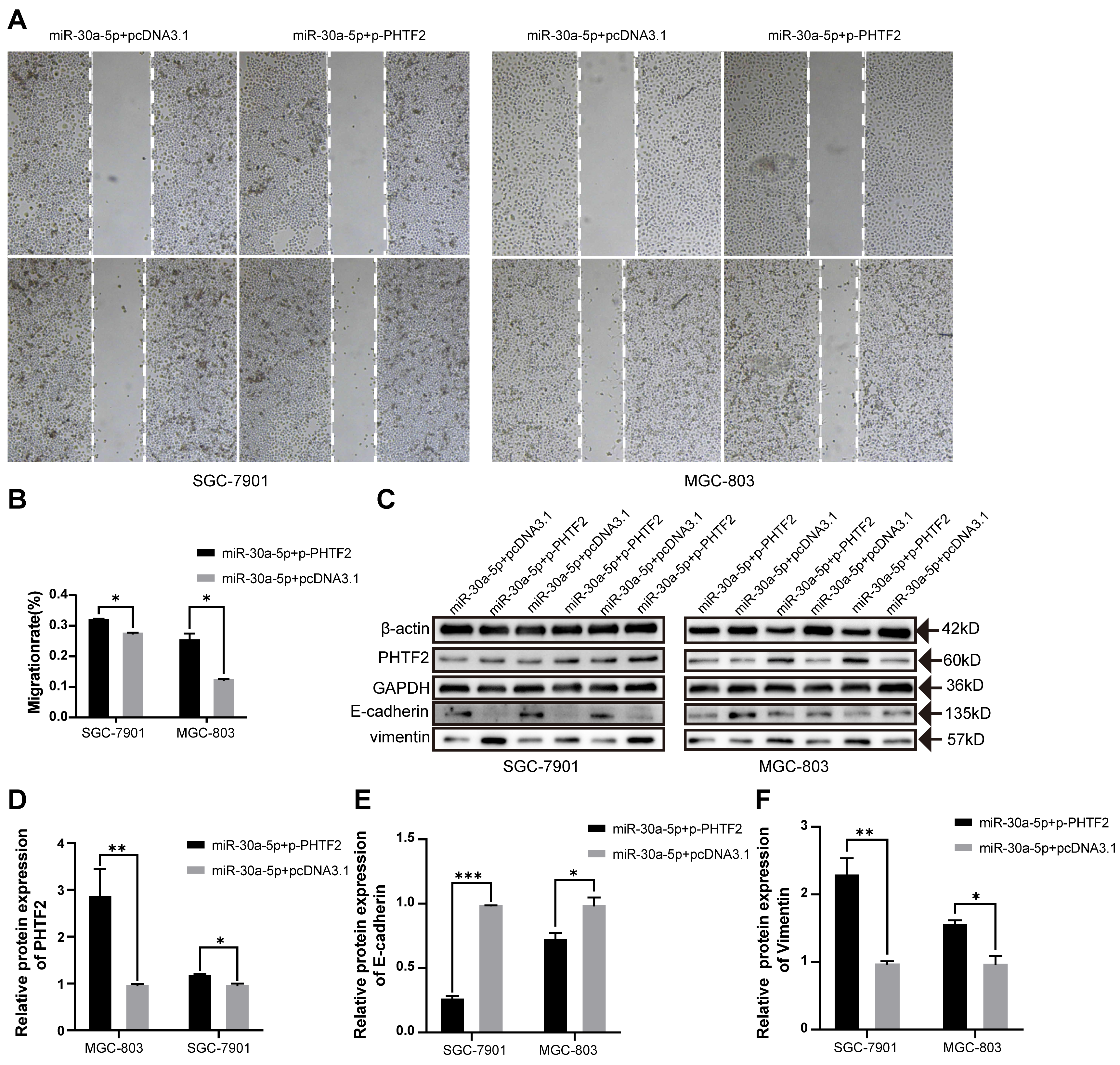

Supplement: Supplementary file 7 — Supplementary Material 7 [file 41598_2025_33375_MOESM7_ESM.tif]
