# Supplementary material for: MiR-30a-5p activates the AKT signalling pathway by targeting PHTF2 to inhibit migration and EMT of gastric cancer
Source: Sci Rep. 2025 Dec 20;16:3401. doi: 10.1038/s41598-025-33375-y (PMC12835005; doi:10.1038/s41598-025-33375-y)

**Fig.6** 7901 miR-30a-5p NC

GAPDH


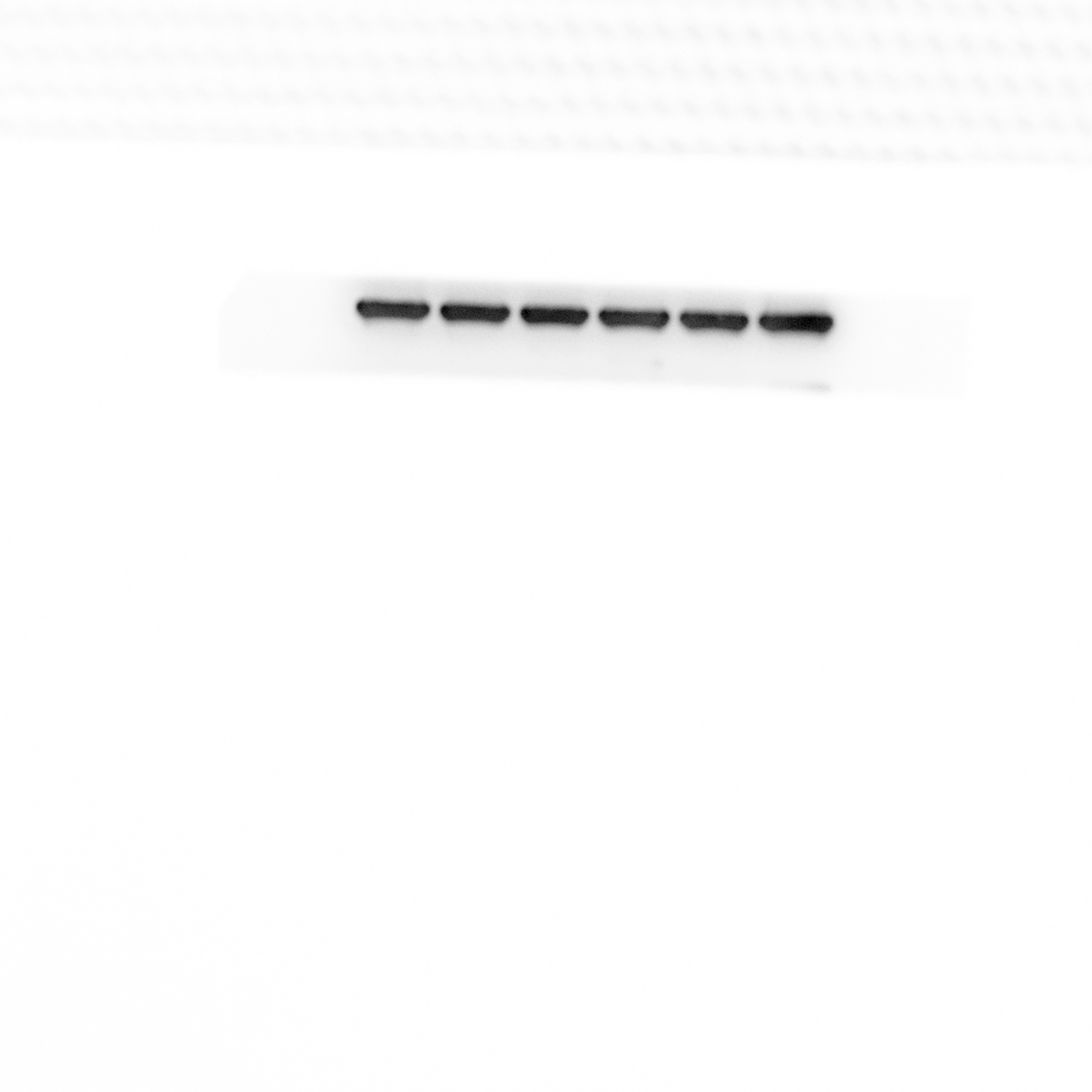


AKT


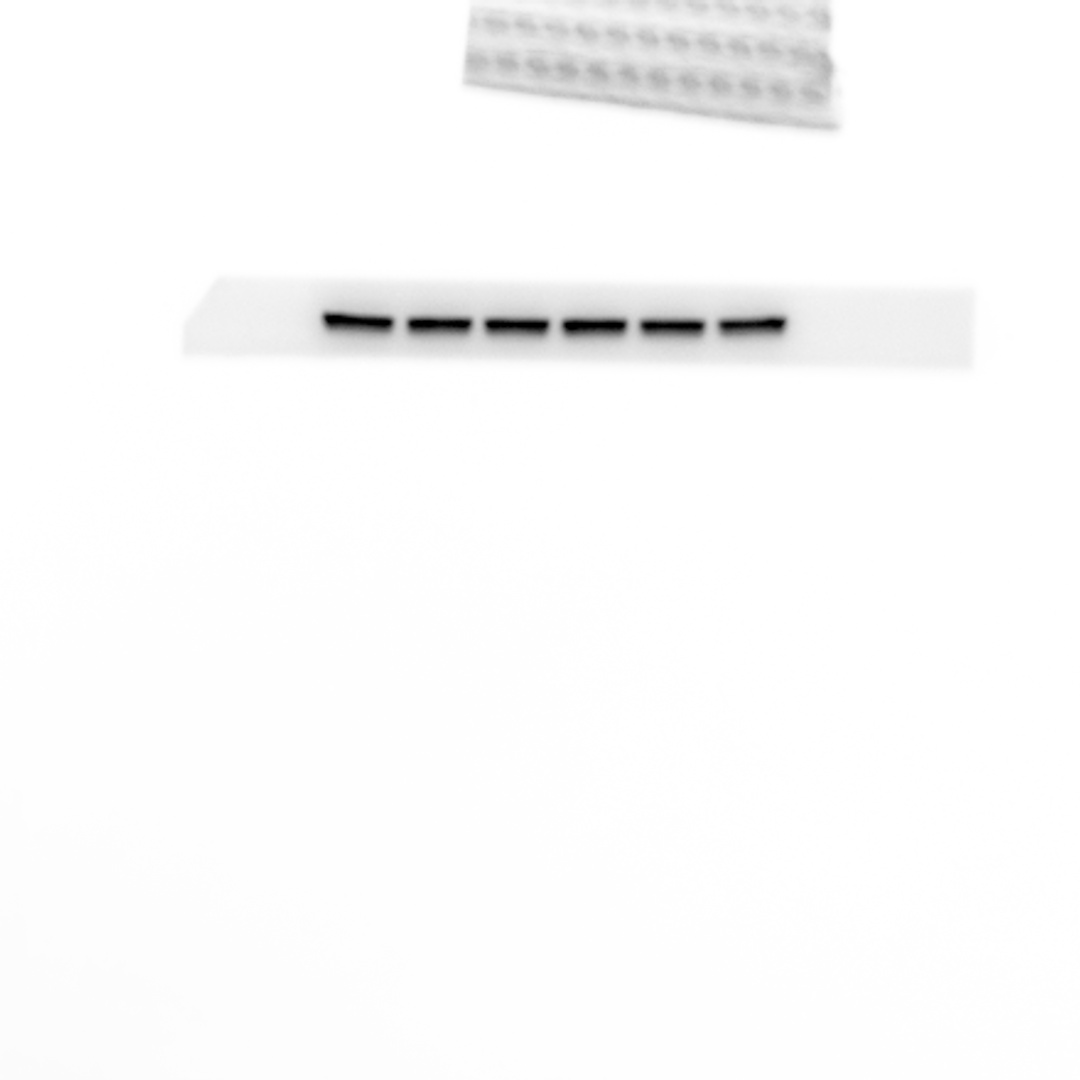


p-AKT


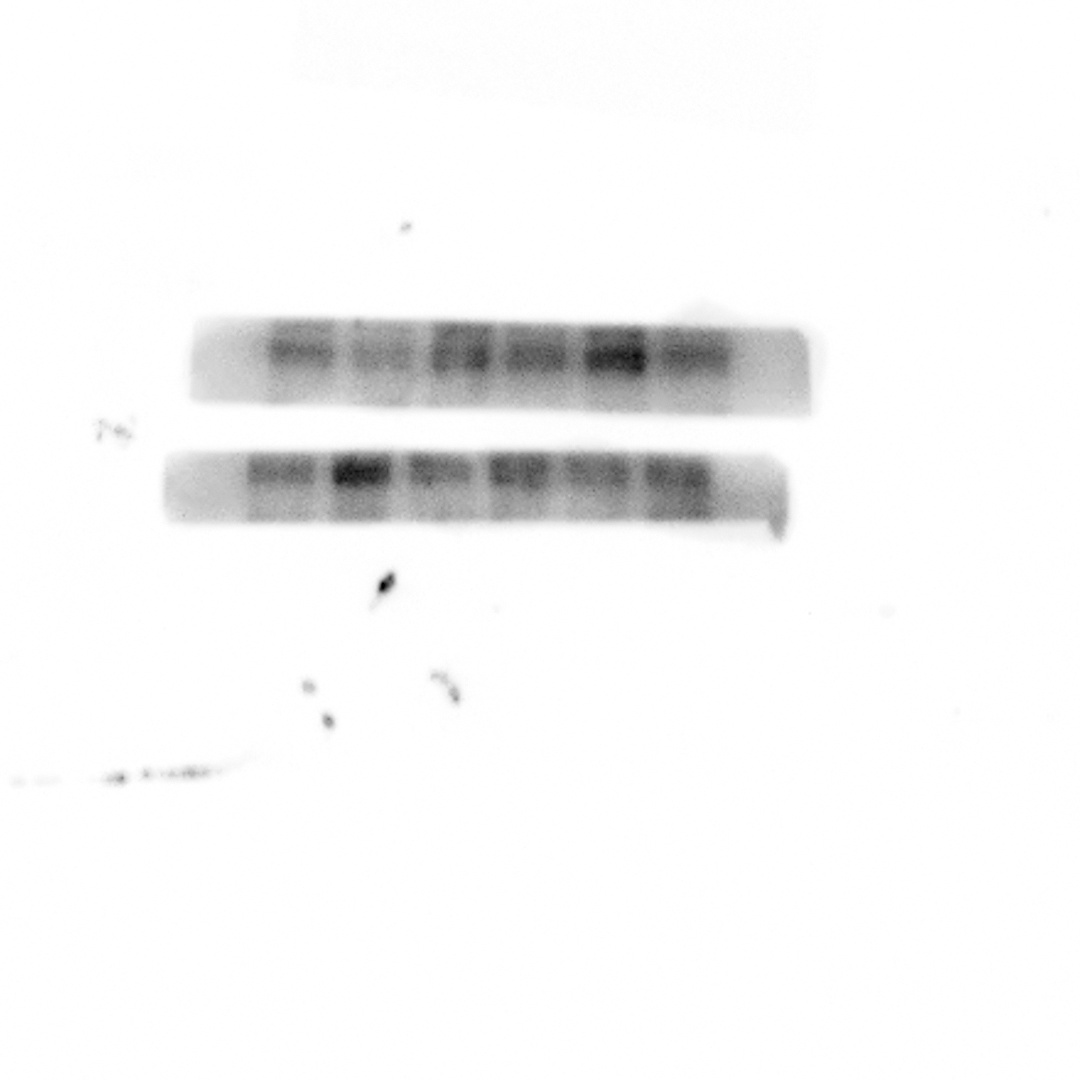


**Fig.6** 7901 miR-30a-5p inhibitor inhibitor NC

GAPDH


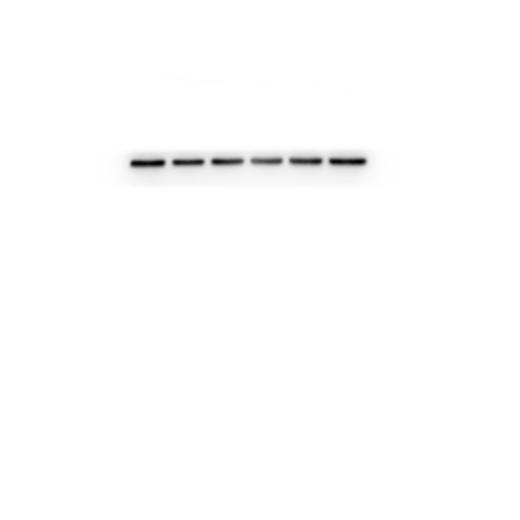


AKT


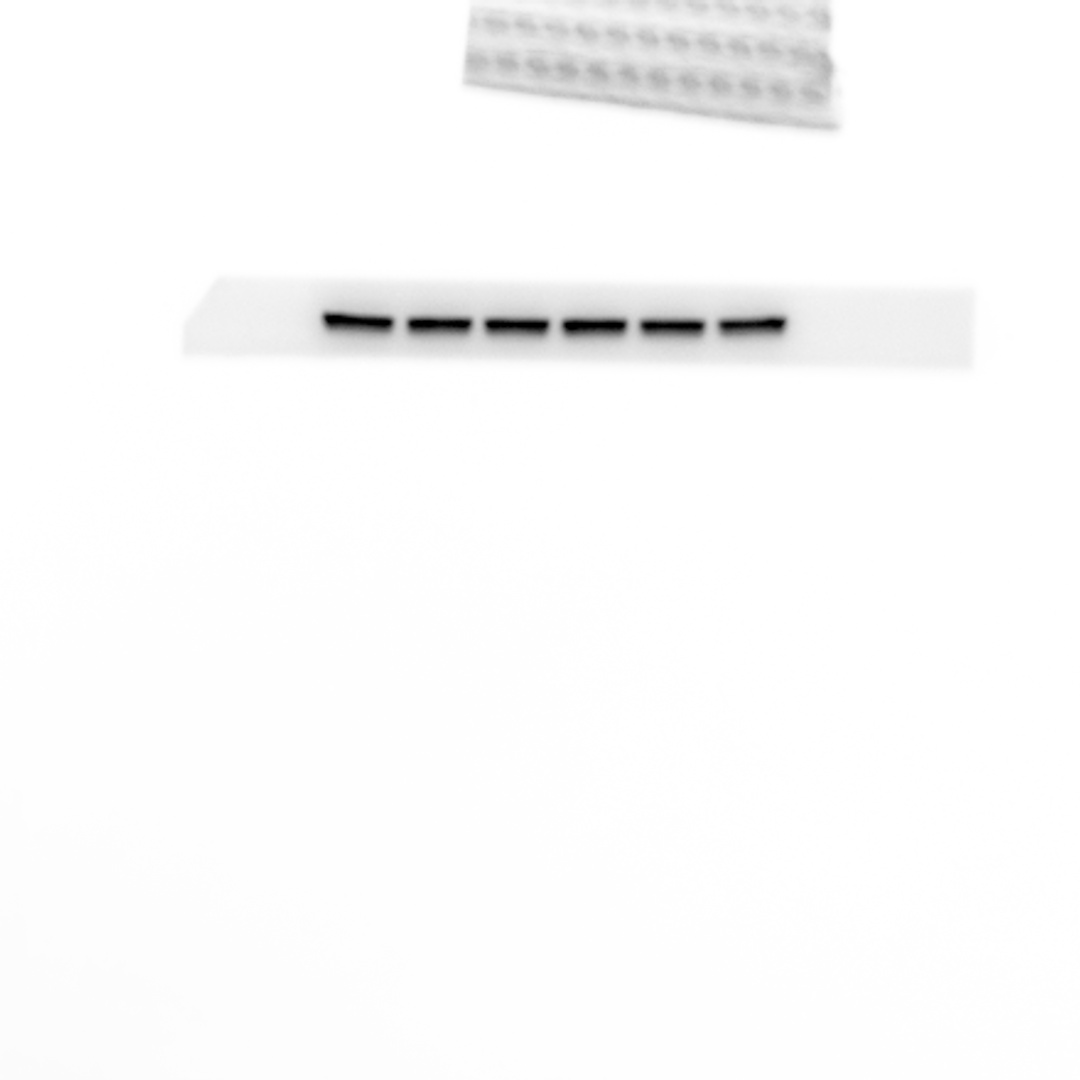


p-AKT


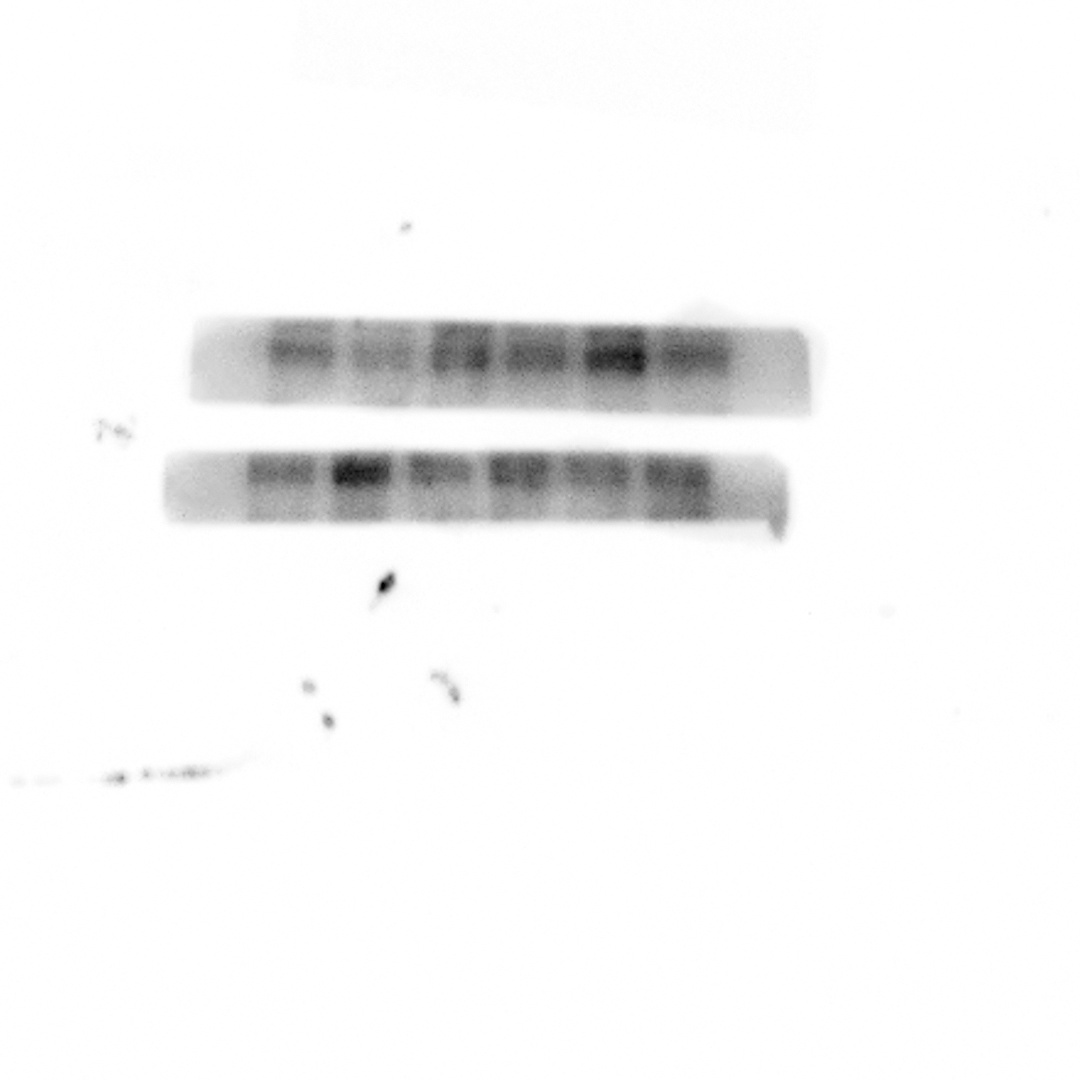


**Fig.6** 7901 si429 si1485 siNC

GAPDH


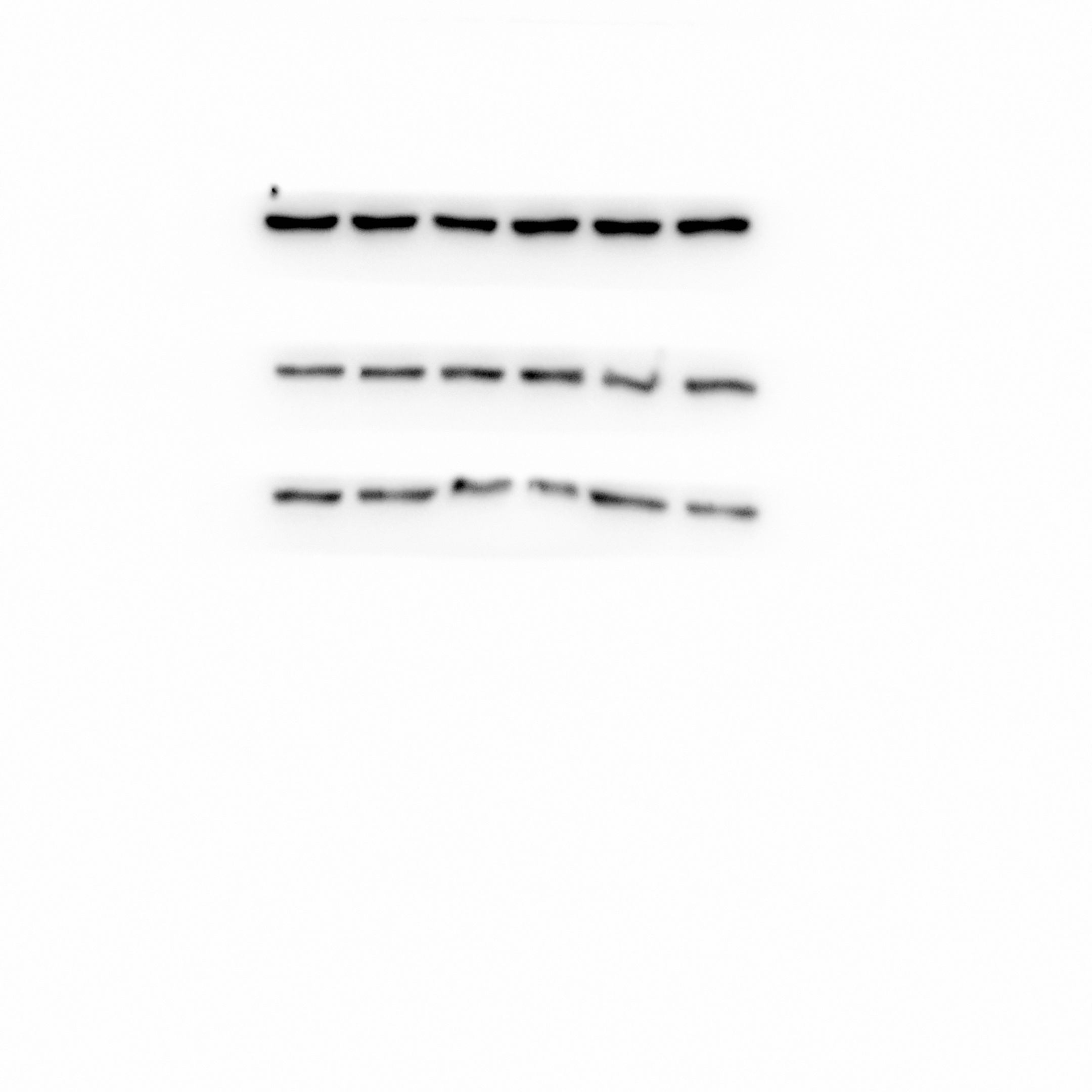


AKT


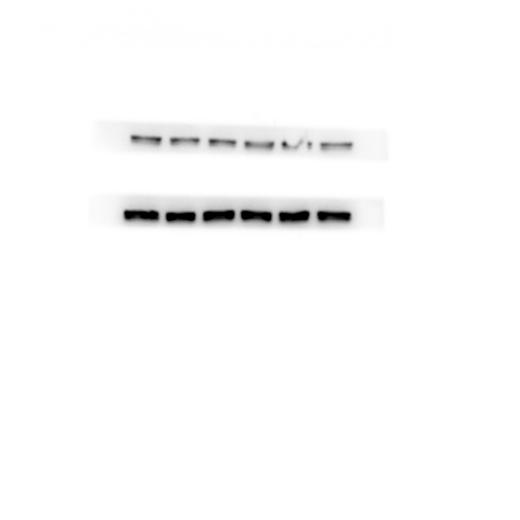


p-AKT


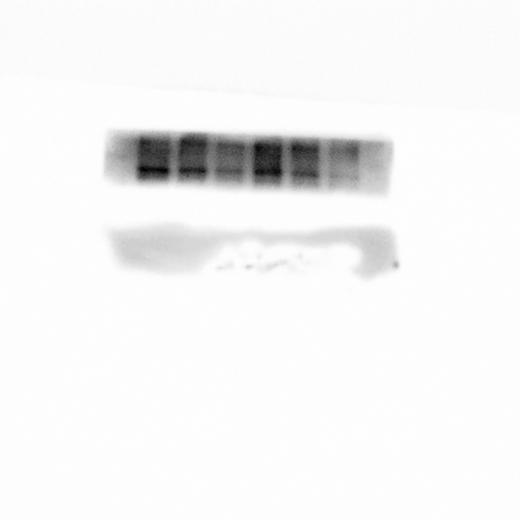


**Fig.6** 7901 pcDNA3.1 p-PHTF2

GAPDH


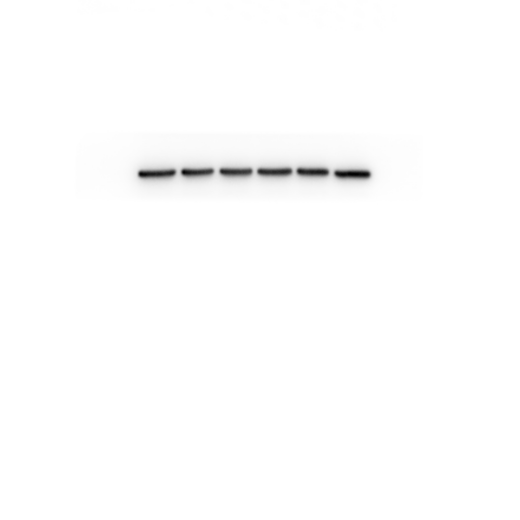


AKT


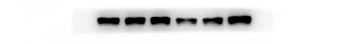


p-AKT


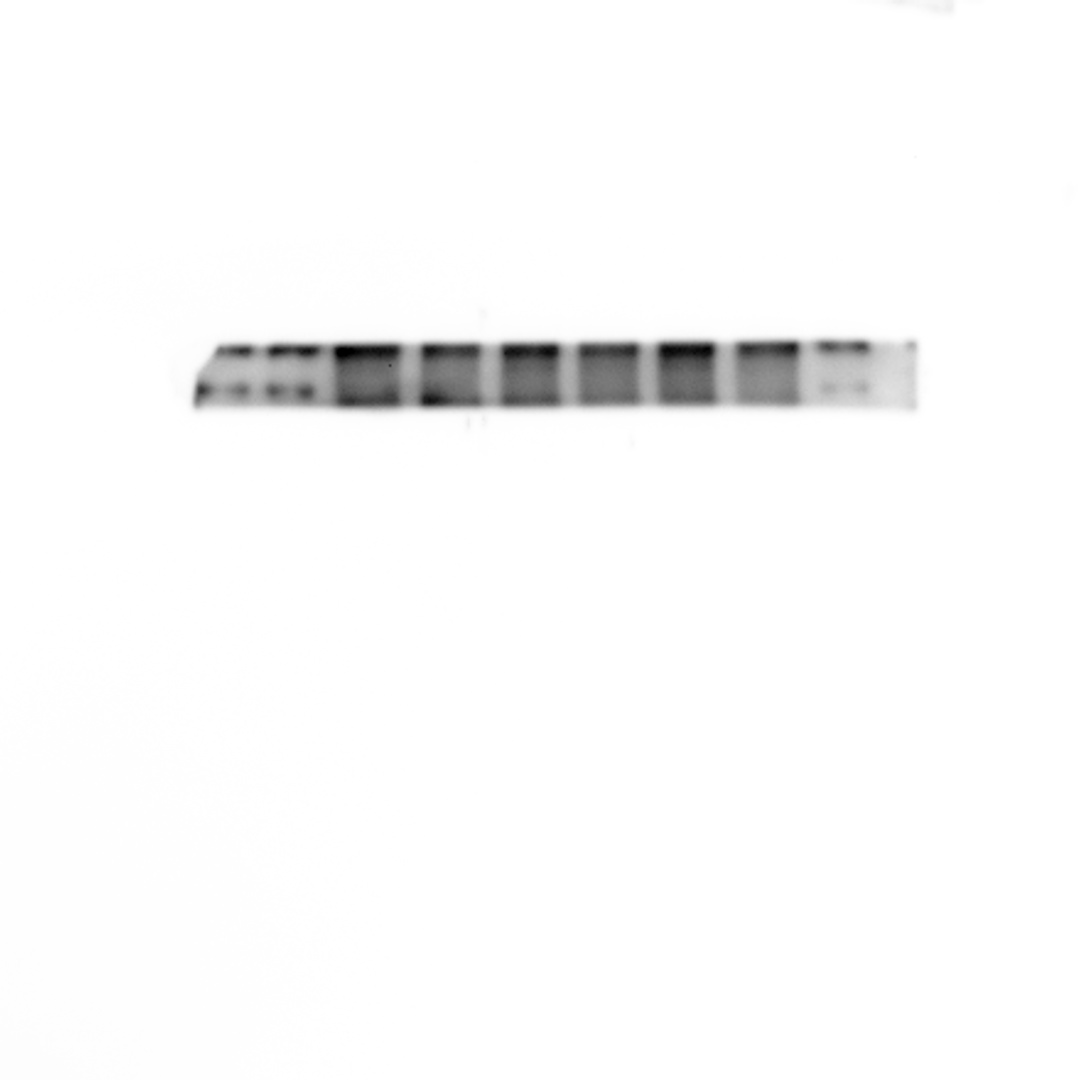


**Fig.6** 803 miR-30a-5p NC

GAPDH


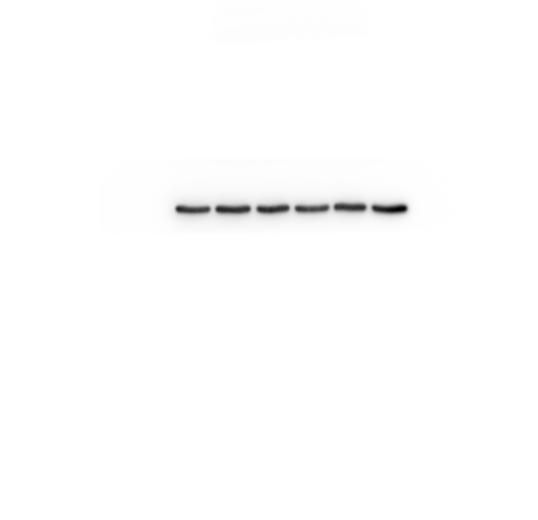


AKT


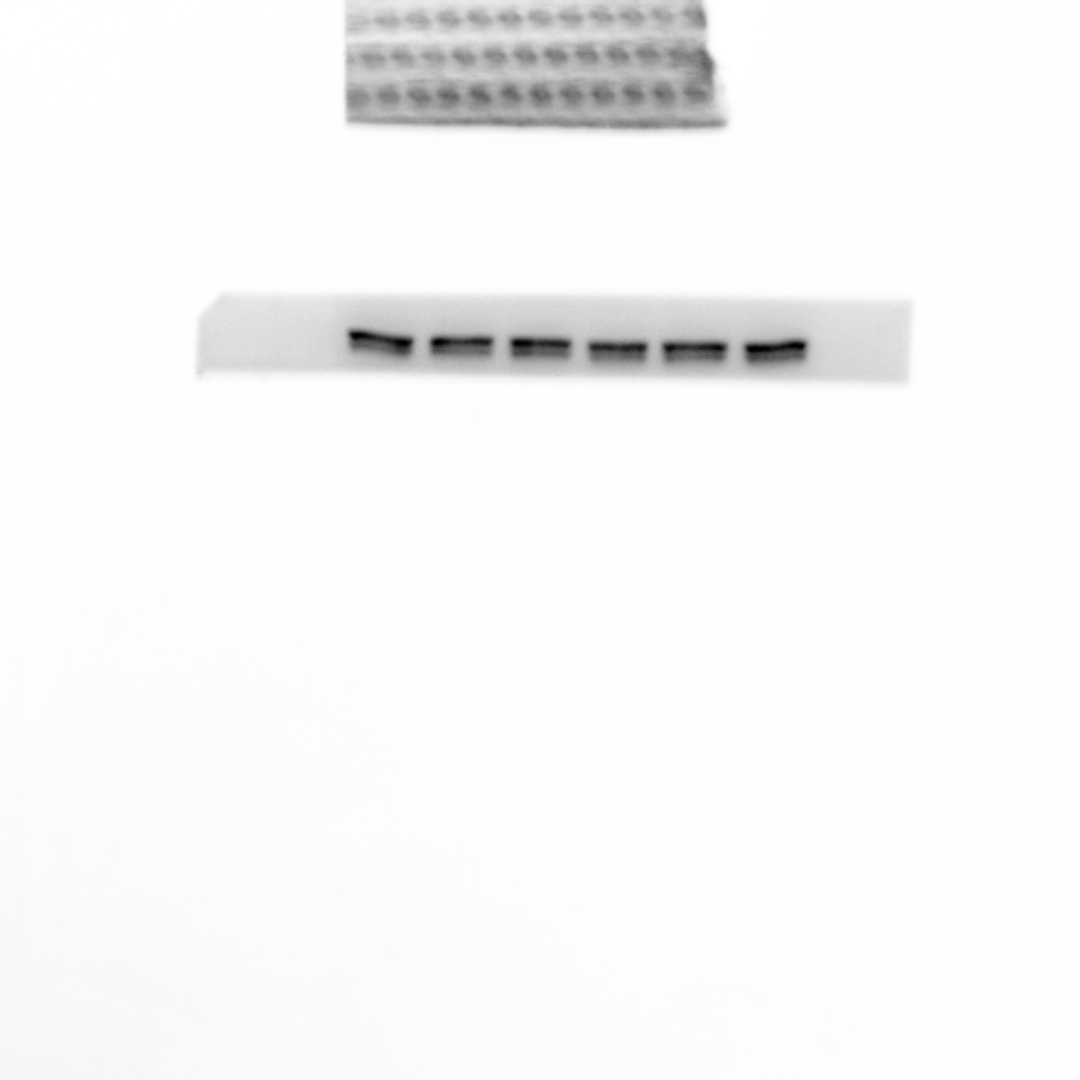


p-AKT


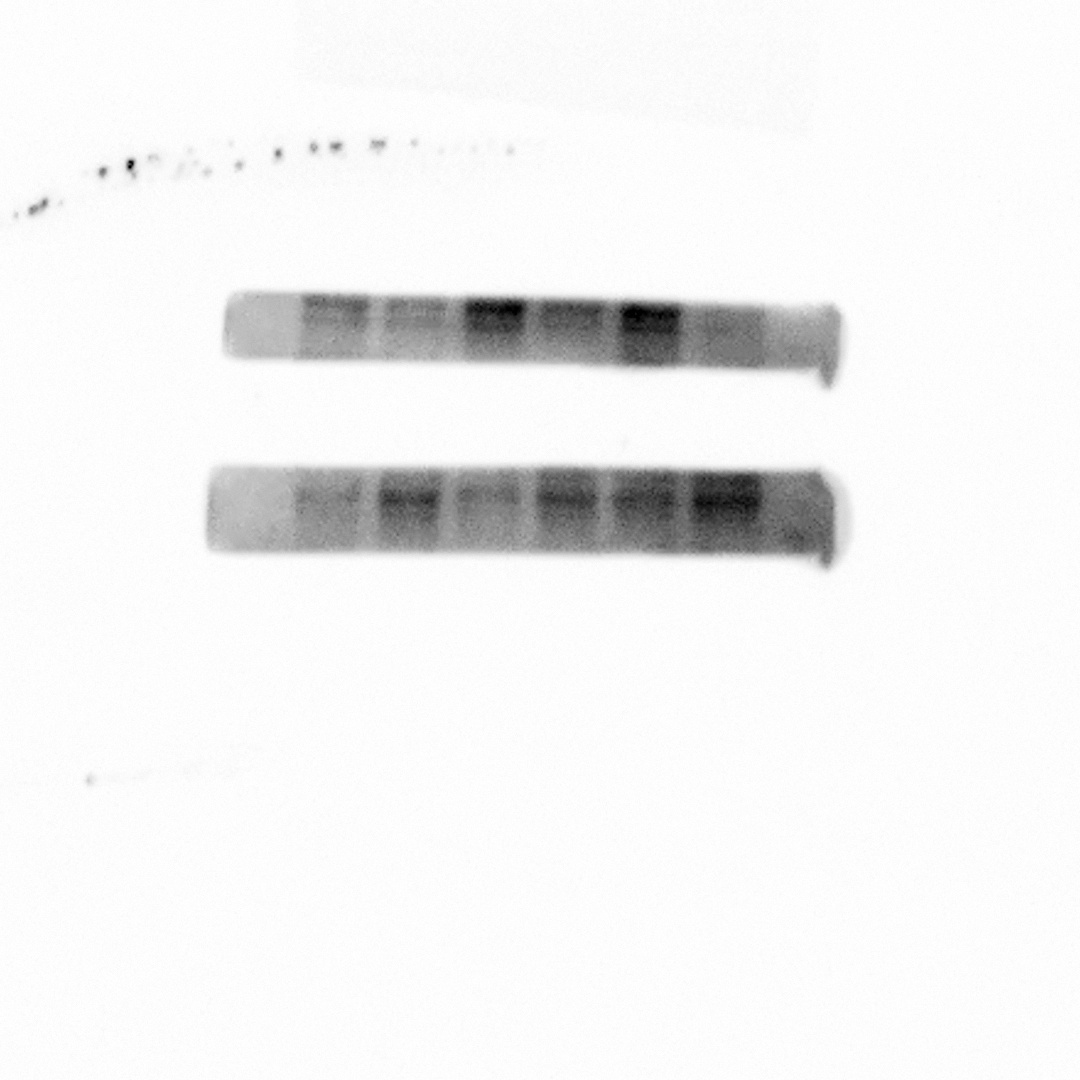


**Fig.6** 803 miR-30a-5p inhibitor inhibitor NC

GAPDH


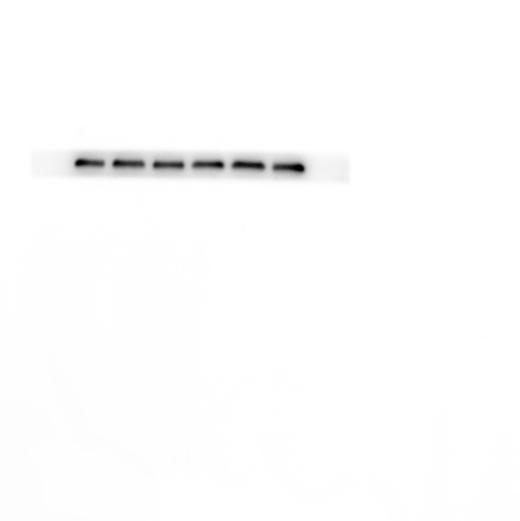


AKT


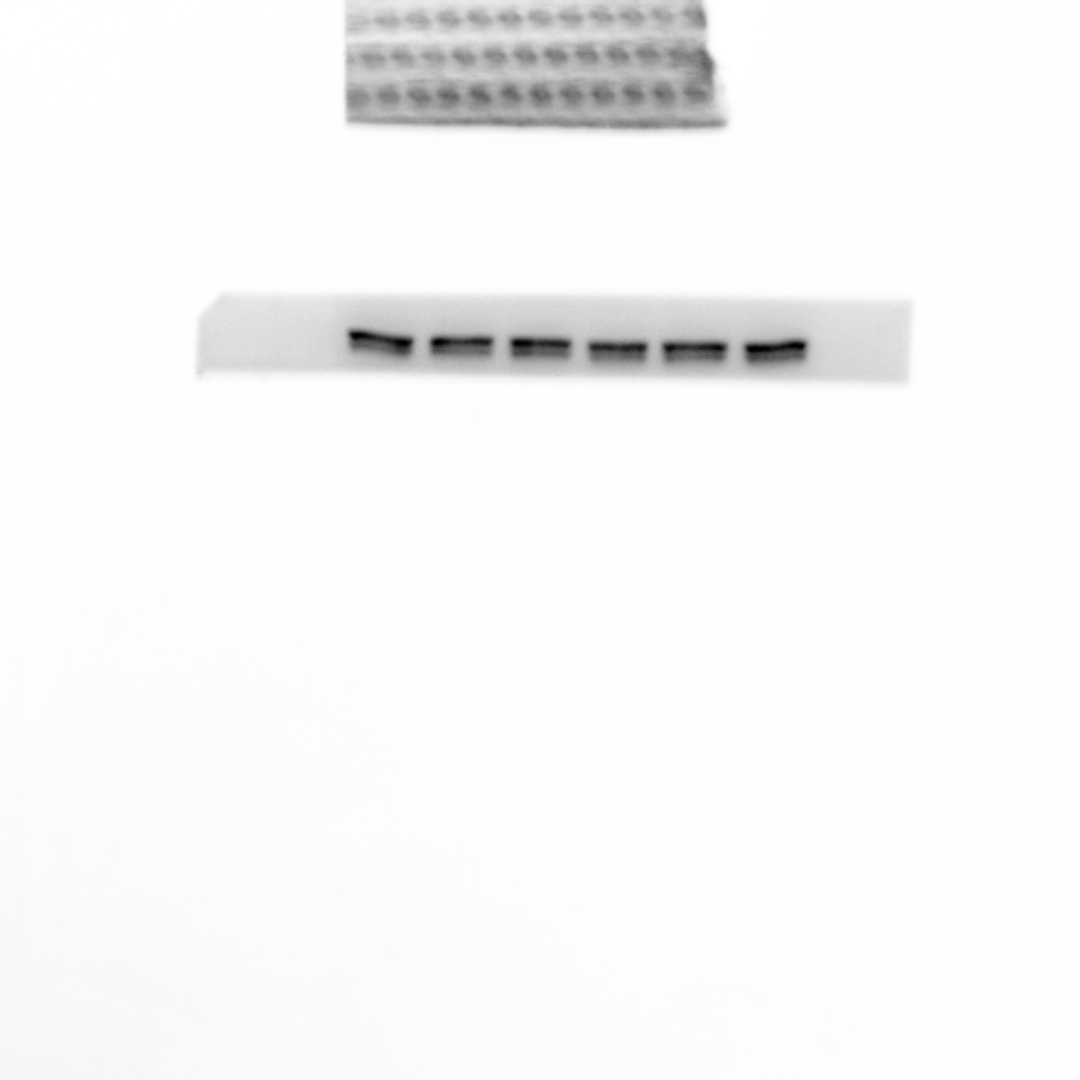


p-AKT


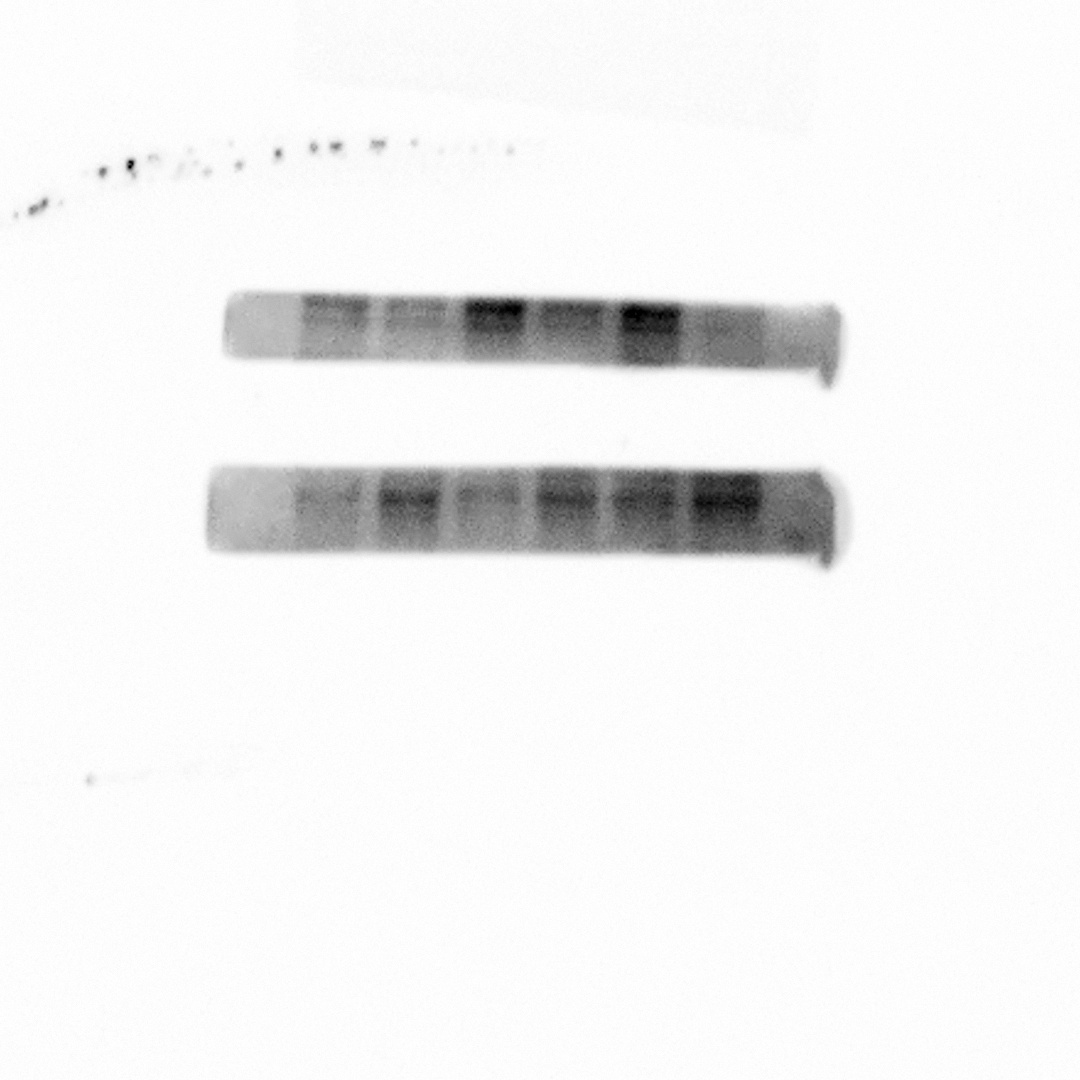


**Fig.6** 803 si429 si1485 siNC

GAPDH


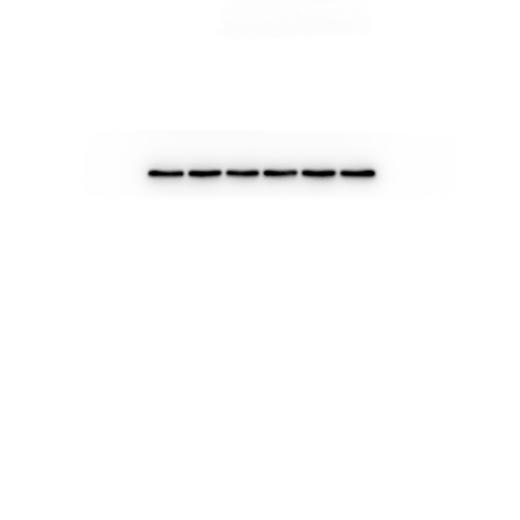


AKT


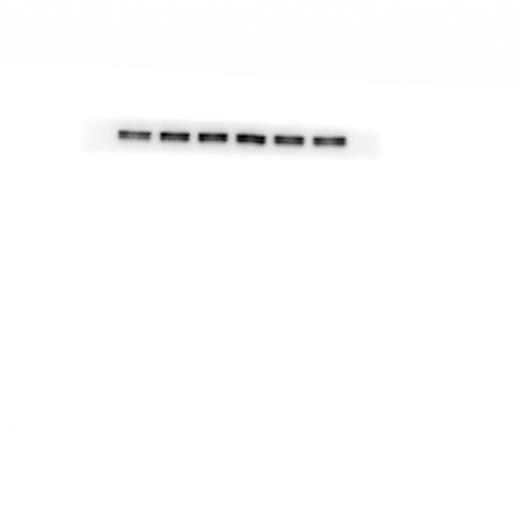


p-AKT


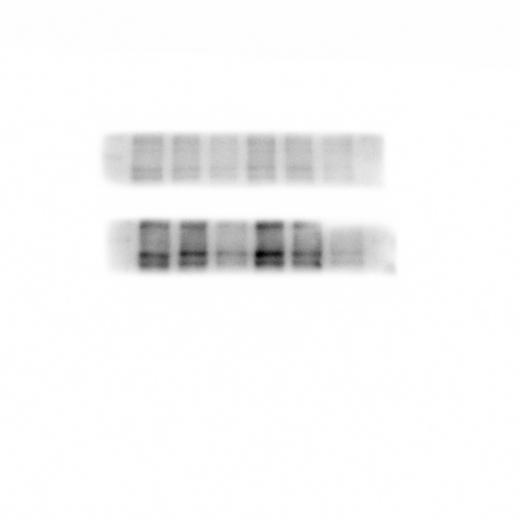


**Fig.6** 803 p-PHTF2 pcDNA3.1

GAPDH


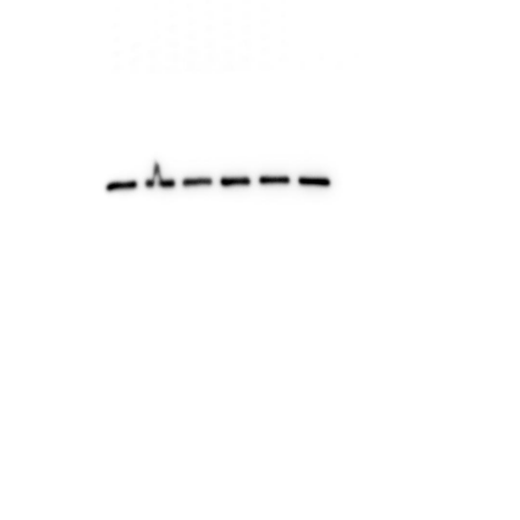


AKT


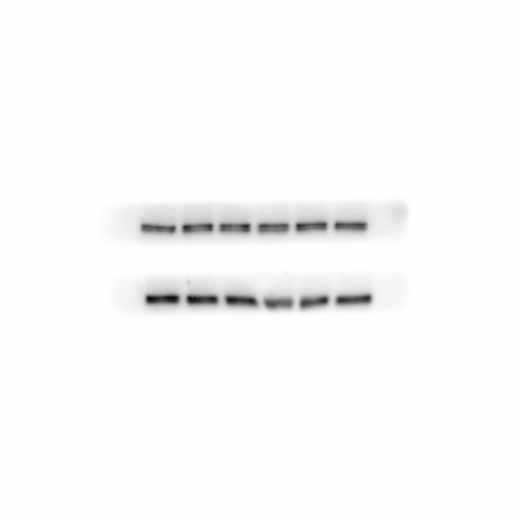


p-AKT


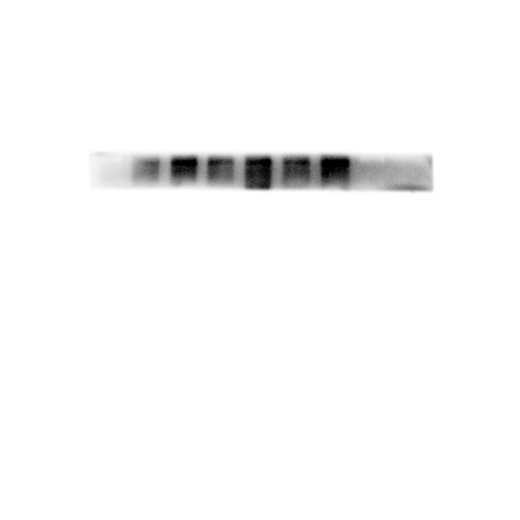

Supplement: Supplementary file 5 — Supplementary Material 5 [file 41598_2025_33375_MOESM5_ESM.docx]
