# Supplementary material for: MiR-30a-5p activates the AKT signalling pathway by targeting PHTF2 to inhibit migration and EMT of gastric cancer
Source: Sci Rep. 2025 Dec 20;16:3401. doi: 10.1038/s41598-025-33375-y (PMC12835005; doi:10.1038/s41598-025-33375-y)

**Fig1S. The expression level of putative targets in SGC-7901.**

1. putative miR-30a-5p target genes by miRDB, miRWalk and mirmap. (B) The expression level of putative targets in SGC-7901 was measured by RT-qPCR. *p < 0.05, **p < 0.01, ns, not significant. NC, negative control. The data expressed as the mean ± SD.

**Fig2S. PHTF2 mediated the biological effects of miR-30a-5p.**

(A-B) The wound healing assays were performed to assess the effect of PHTF2 in miR-30a-5p-overexpressing cells on cell motility at 0 and 48h. (C-F) The effect of PHTF2 in miR-30a-5p-overexpressing cells on E-cadherin and vimentin protein expression in MGC-803 and SGC-7901 cells was detected by Western blot. *p < 0.05, **p < 0.01, ***p < 0.001. The data expressed as the mean ± SD.

Fig2S-01

SGC-7901：

β-actin


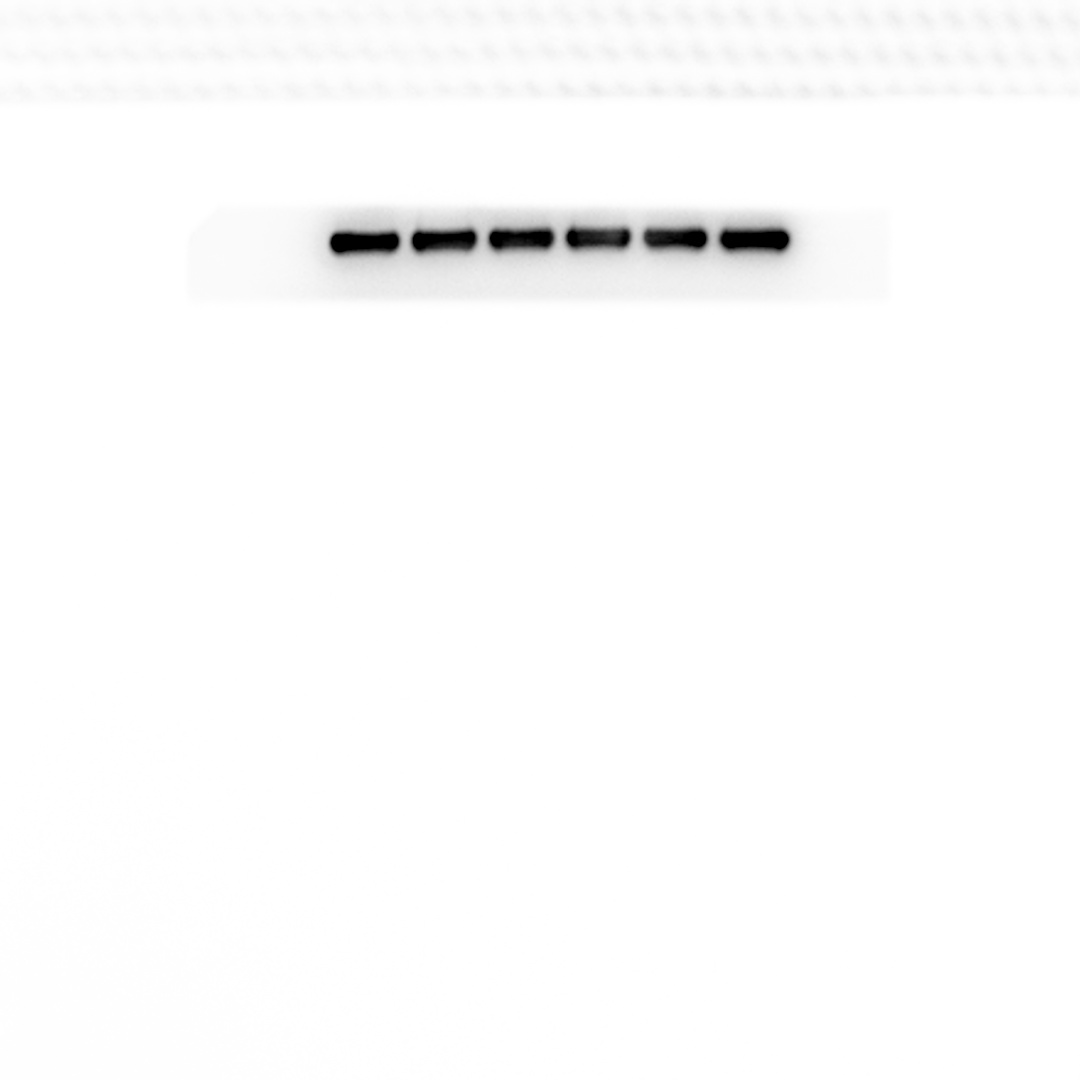


PHTF2


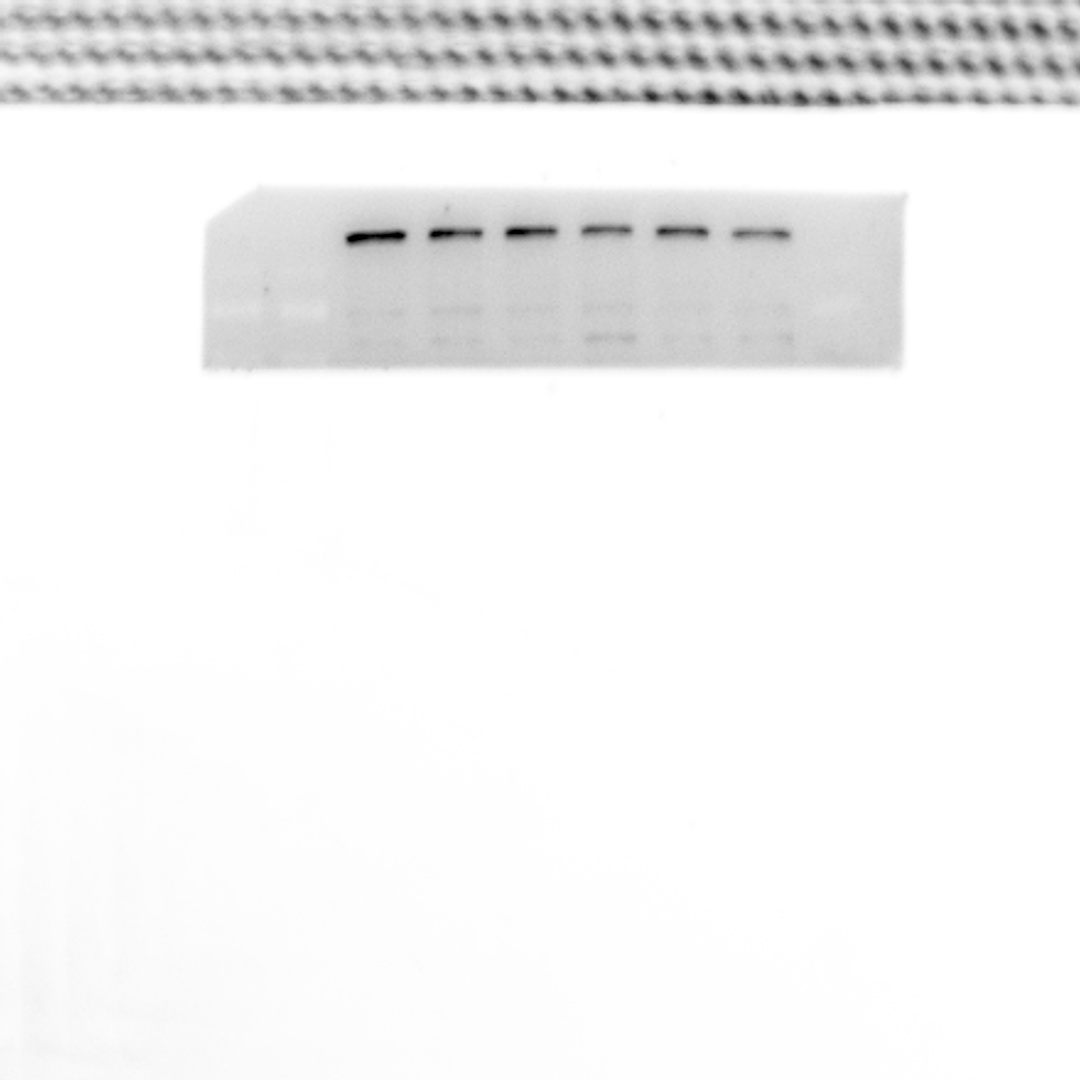


GAPDH


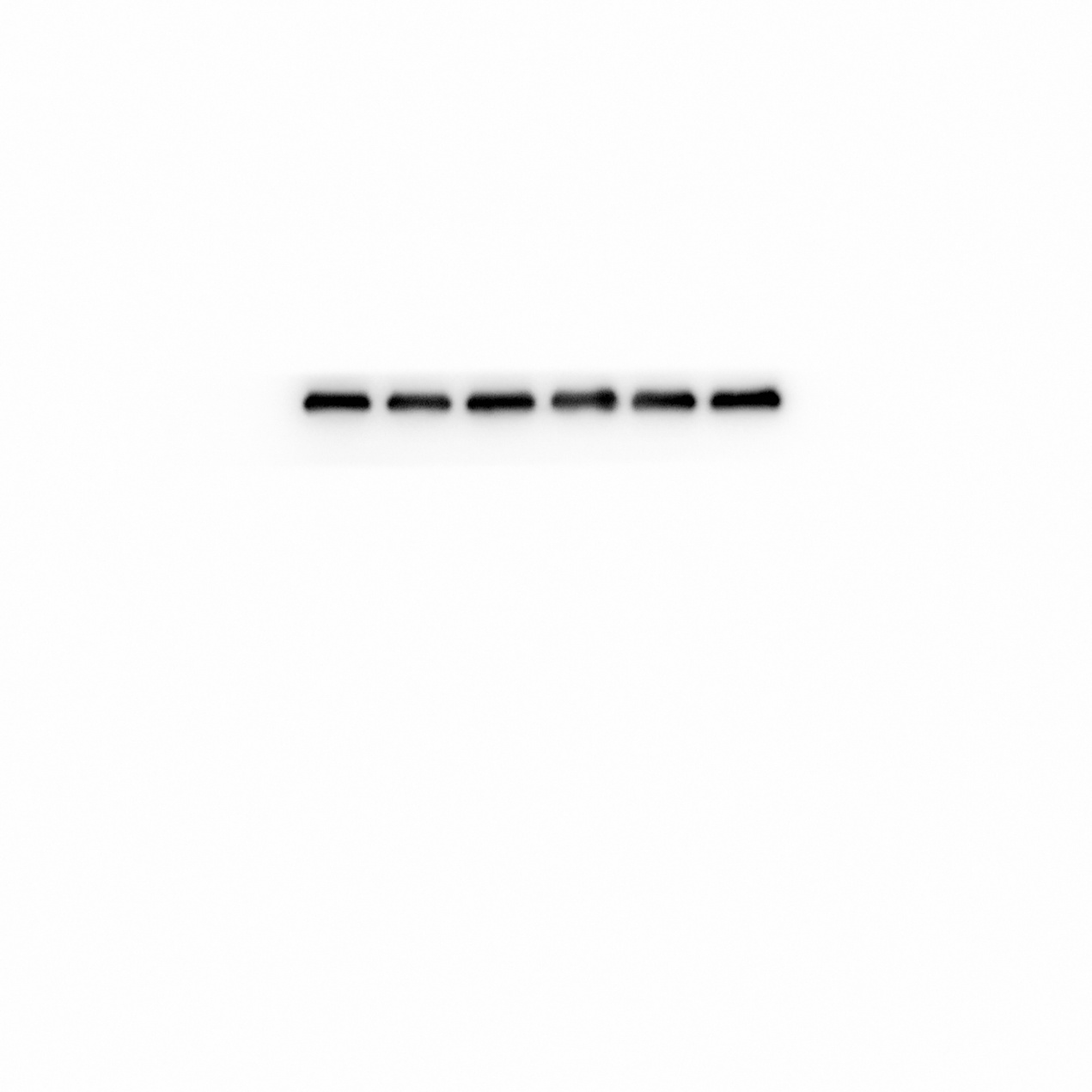


E-cadherin


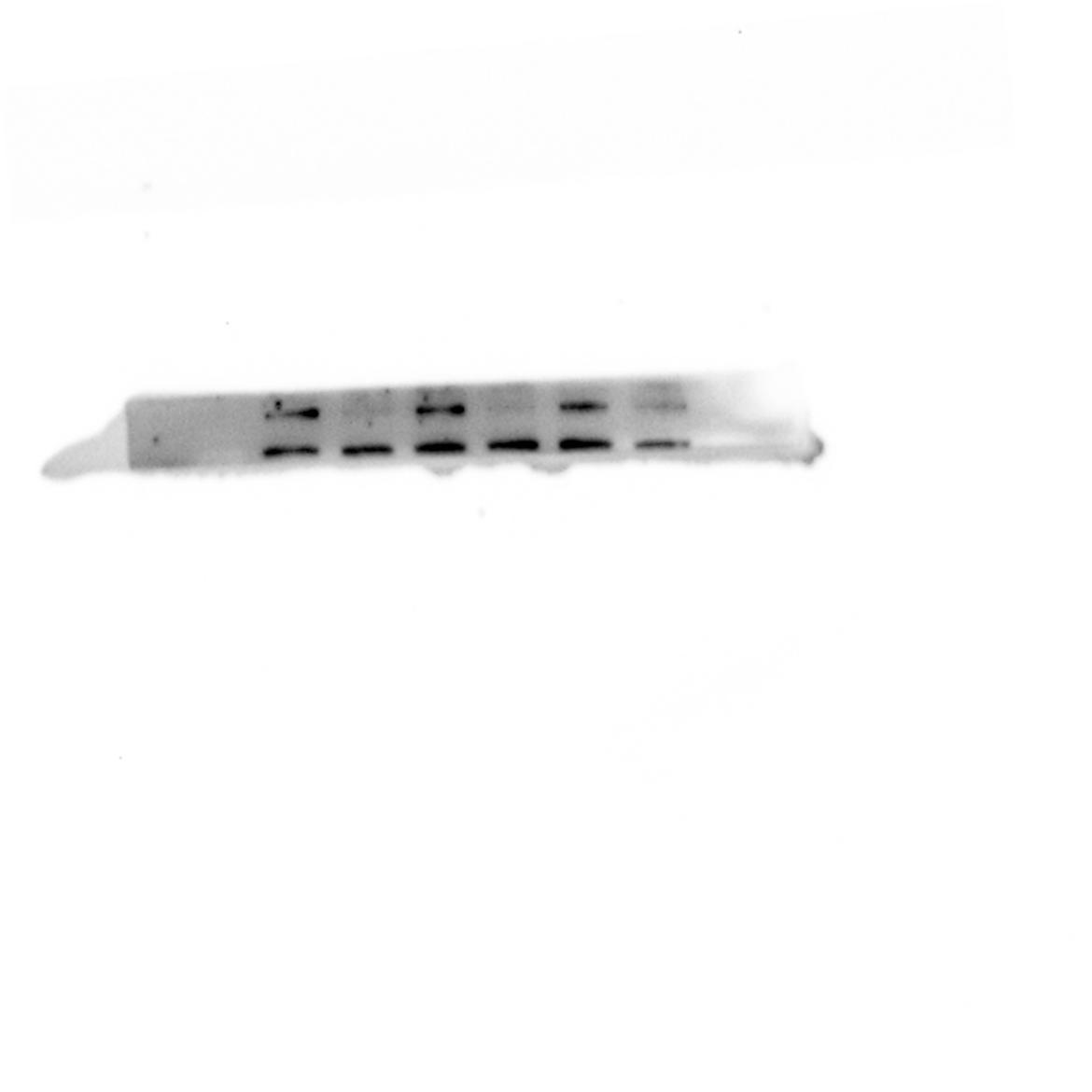


Vimentin


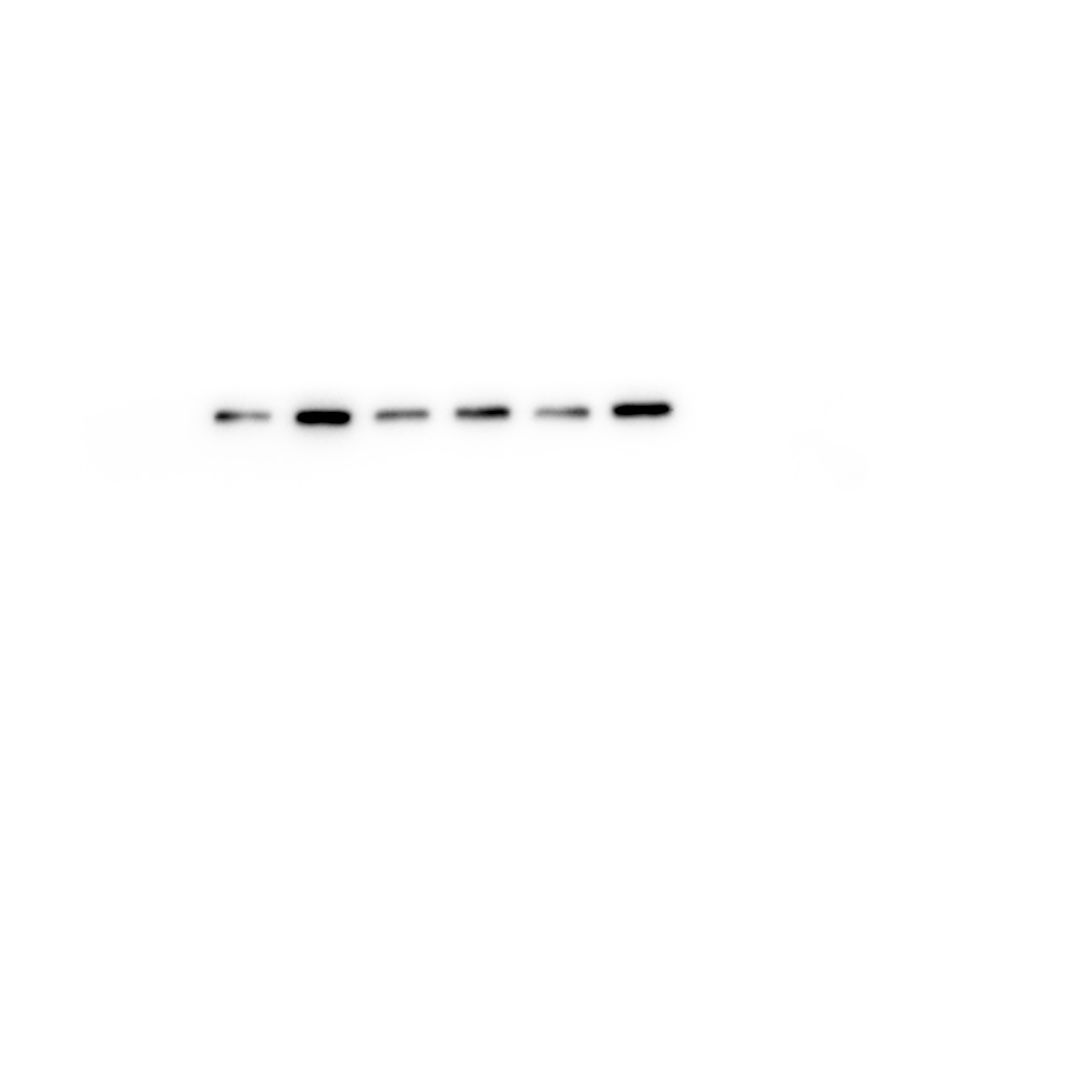


MGC-803：β-actin





PHTF2





GAPDH


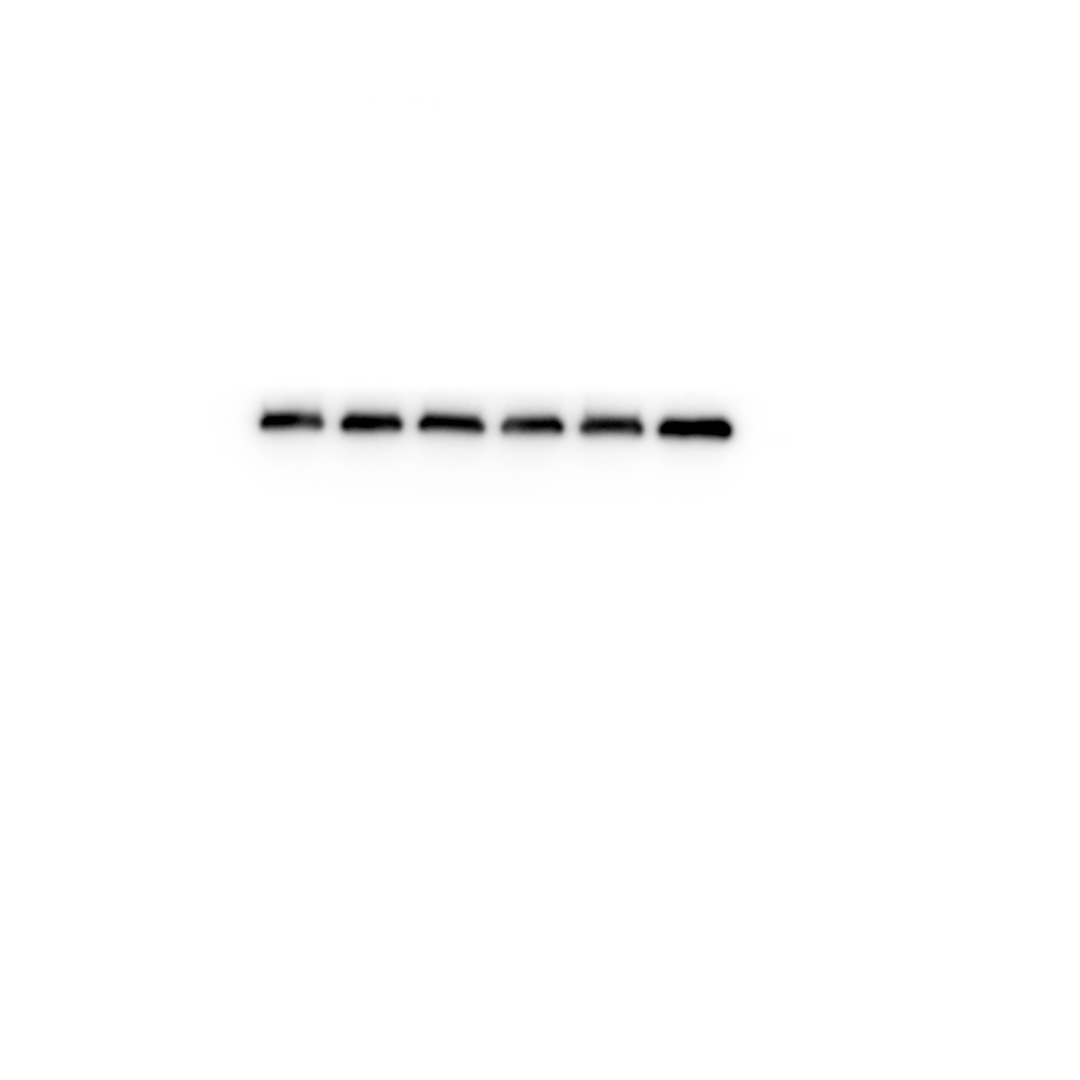


E-cadherin


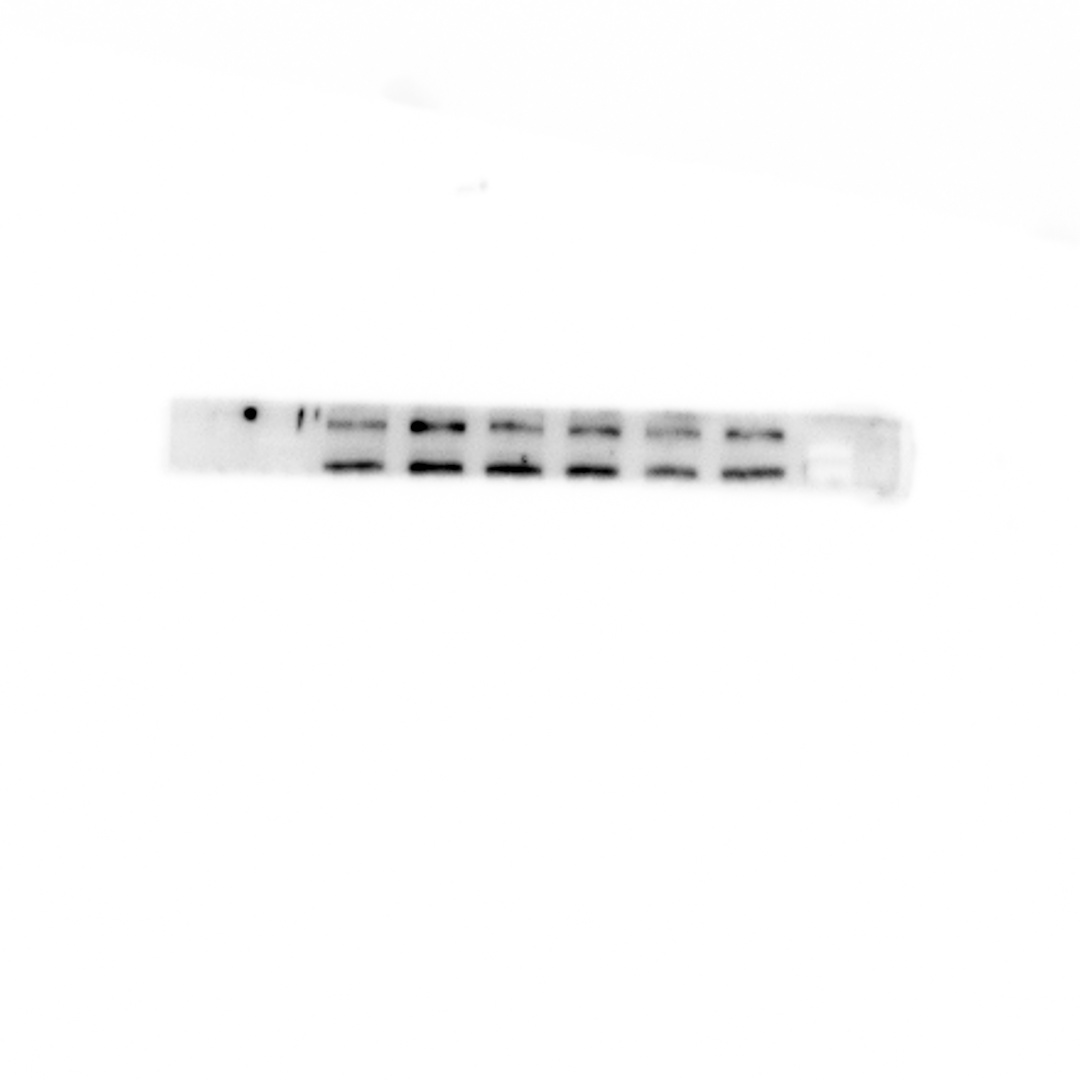


Vimentin


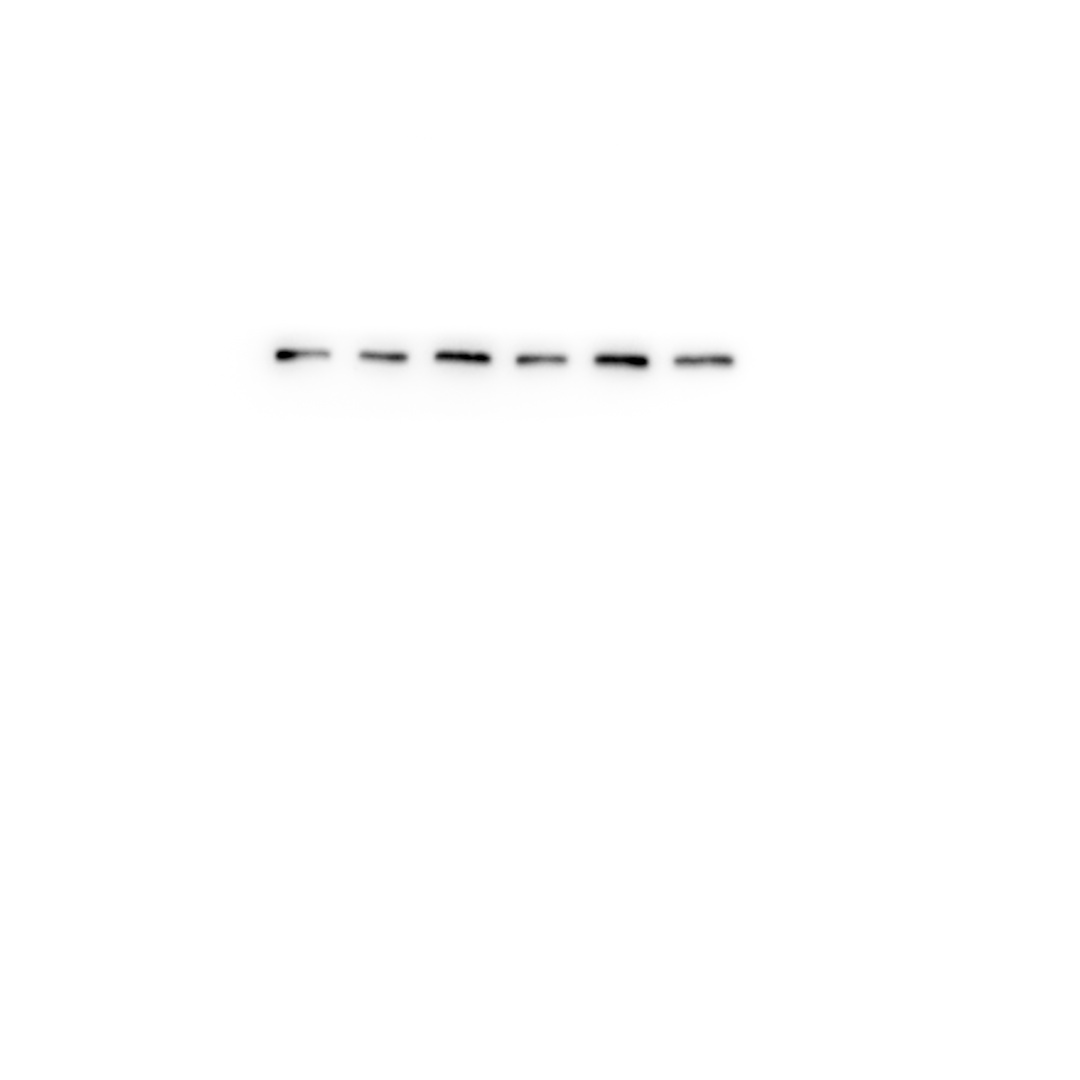

Supplement: Supplementary file 8 — Supplementary Material 8 [file 41598_2025_33375_MOESM8_ESM.docx]
